# The Impact of Recipient HIV Status on Kidney Transplant Outcomes in the United Kingdom: Have Previous Studies Overestimated Risk?

**DOI:** 10.1097/TP.0000000000005585

**Published:** 2025-12-03

**Authors:** Marena L. Gray, Samuel J. Tingle, Mohamed Elzawahry, Eliot Hurn, Balaji Mahendran, Frank A. Post, Rachel Hilton

**Affiliations:** 1 Department of Surgery, West Suffolk Foundation Trust, Bury St Edmunds, United Kingdom.; 2 Translational and Clinical Research Institute, Newcastle University, Newcastle upon Tyne, United Kingdom.; 3 Nuffield Department of Surgical Sciences, University of Oxford, Oxford, United Kingdom.; 4 Department of Sexual Health, Lewisham and Greenwich NHS Foundation Trust, London, United Kingdom.; 5 Department of Surgery, Royal Cornwall Hospitals NHS Trust, Truro, United Kingdom.; 6 Department of Sexual Health and HIV, King’s College Hospital NHS Foundation Trust, Weston Education Centre (2.53), London, United Kingdom.; 7 Directorate of Transplant, Renal and Urology, Guy’s Hospital, Guy’s and St. Thomas’ NHS Foundation Trust, London, United Kingdom.

## Abstract

**Background.:**

Despite increasing literature on kidney transplantation in people with HIV, studies have largely overlooked the confounding impact of ethnicity and socioeconomic deprivation. This study aimed to assess posttransplant outcomes in HIV-positive recipients, adjusting for these key factors.

**Methods.:**

Population-cohort study of single kidney-alone transplants (2005-2022) using the UK Transplant Registry. The primary outcome was graft survival. Multivariable regression models evaluated the impact of recipient HIV status on transplant outcomes.

**Results.:**

We included 30 013 recipients: 20 517 deceased-donor (200 HIV-positive recipients) and 9496 live-donor (44 HIV-positive recipients). Multivariable models revealed no significant differences in 5-y graft survival for HIV-positive versus negative recipients, in either deceased (adjusted hazard ratio = 0.93, 95% confidence interval 0.61-1.41, *P* = 0.721) or live donor cohorts (adjusted hazard ratio = 1.02 [0.31-3.33]; *P* = 0.973). Recipient HIV status was also not associated with patient survival or delayed graft function in either cohort. HIV-positive recipients were more likely to experience acute rejection within 12 mo (deceased adjusted odds ratio = 1.53 [0.94-2.50]; *P* = 0.091 and live adjusted odds ratio = 3.43 [1.44-8.19]; *P* = 0.006) and had marginally lower adjusted 12-mo estimated glomerular filtration rate. Crude 12-mo acute rejection rates for HIV-positive versus negative recipients were 24.8% versus 14.2% (deceased) and 37.5% versus 15.3% (live). For all posttransplant outcomes, removing adjustment for ethnicity and deprivation led to greater adjusted risk estimates for HIV-positive recipients.

**Conclusions.:**

HIV-positive recipients achieve excellent graft and patient survival, which are no different from HIV-negative recipients on adjusted analyses. Previous studies without ethnicity or socioeconomic-deprivation adjustment have likely overestimated the risks of recipient HIV-positive transplants. HIV-positive recipients with an indication for kidney transplantation should have equitable access to assessment, listing and offers for kidney transplantation.

## Graphical abstract

**Figure:**
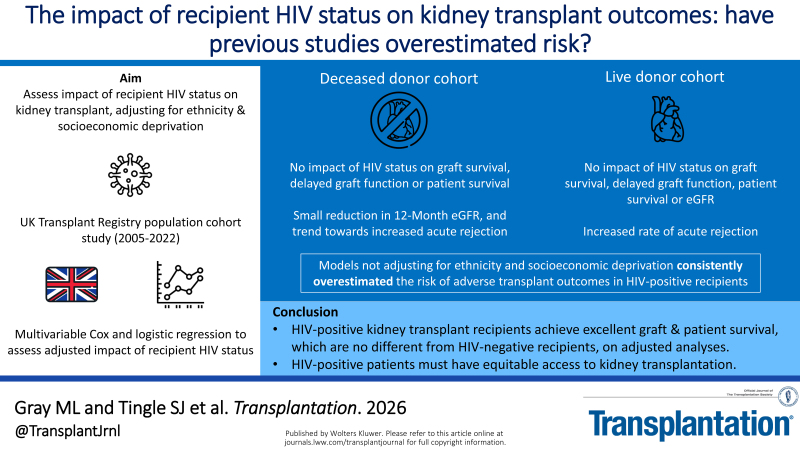

## INTRODUCTION

People living with HIV, particularly those of Black ethnicity, are at an increased risk of end-stage kidney disease, with HIV-associated nephropathy and other renal complications contributing to morbidity and mortality in this group.^1-4^ The success of combination antiretroviral therapy (cART) has improved survival for HIV patients, leading to growing numbers of individuals with HIV pursuing kidney transplantation.^5-7^ However, this remains complex, as increased rates of graft rejection have been reported in HIV-positive recipients, and HIV-induced immune dysfunction may increase the risk of infection in the setting of transplant-immunodeficiency. In addition, patients who have experienced virologic failure on previous regimens may require a boosted protease inhibitor regimen to achieve better viral suppression, in which case careful coordination with immunosuppressive regimens is necessary to minimize interactions and toxicity.^5^

Several studies, ranging from the US Scientific Registry of Transplant Recipients and European cohorts, have demonstrated comparable graft and patient survival in HIV-positive and HIV-negative recipients.^8-11^ UK-specific data also suggest favorable outcomes, although these are based on relatively smaller cohorts.^7,12^ Importantly, most of these studies have not accounted for the impact of ethnicity or relative socioeconomic deprivation, both of which are known to independently influence posttransplant outcomes.^7,9,13-15^ Additionally, immunosuppression regimes vary internationally: the United States commonly employs anti-thymocyte globulin for induction, whereas interleukin-2 receptor antagonists (IL2RA) are favored in the United Kingdom, reflecting divergent clinical practices and underlying uncertainty about optimal approaches in this population.^7,16-18^

To date, few studies have examined kidney transplant outcomes in individuals living with HIV while simultaneously adjusting for ethnicity and relative socioeconomic deprivation. This study aims to assess the impact of recipient HIV status on posttransplant outcomes, while adjusting for these important confounders. We also aim to assess whether previous studies that have not adjusted for recipient ethnicity and socioeconomic deprivation will have overestimated the risks of HIV-positive recipient transplant.

## MATERIALS AND METHODS

This was a national population-cohort study from all 23 UK kidney transplant centers, using retrospectively collected data from the UK National Health Service Blood and Transplant (NHSBT) Registry. This is a computer-based record of all organ donors, organ recipients and those on the waiting list for transplant. Personal and clinical details are collected at the time of registration for a transplant, time of the transplant operation, postoperatively, and at set follow-up intervals to help with effective allocation, monitoring, and to ensure equitable access to transplantation services.^19^

Recipient and donor sex are reported as recorded in the UK NHSBT Transplant Registry. Ethnicity is reported exactly as classified by the UK NHSBT Transplant Registry; recipient ethnicity is self-reported and coded based on their registration at their primary care center, and further information is entered during their enrollment onto the transplant waiting list by Specialist Nurses.^20^ Relative deprivation was determined using the index of multiple deprivation (IMD, 2010), which is an official measure of relative deprivation used in the UK based on postcode.^21^ IMD combines information on income, employment, education, health, crime, living environment, and barriers to services. A higher IMD score indicates more socioeconomic deprivation.

We included adult (aged 18 y and over at the time of transplant) recipients of single kidney-alone transplants performed between January 1, 2005, and December 31, 2022. The following were excluded: multiple organ transplants (which included dual organ kidney transplants and simultaneous pancreas-kidney), missing recipient HIV data, and antibody incompatible transplants. This was split into deceased and live donor groups. Data were extracted on April 19, 2024.

The primary exposure was recipient HIV status. The primary outcome was graft survival (censored at death or 5 y). Secondary outcomes were patient survival (censored at 5 y), delayed graft function (DGF; defined as dialysis in the first week following transplantation^22^), 12-mo treated acute rejection (with or without biopsy), and 12-mo graft function (estimated glomerular filtration rate [eGFR]). This was calculated using the Chronic Kidney Disease Epidemiology Collaboration 2021 formula,^23^ using serum creatinine, age, and patient sex, without race. Individuals who lost their graft before 12 mo were given a nominal value of 10 mL/min/1.73 m^2^.^24^

The study was conducted in accordance with the Declaration of Helsinki and approved by the UK NHSBT Registry. This study followed the Strengthening the Reporting of Observational Studies in Epidemiology reporting guideline.^25,26^

### Statistical Analysis

The approach for missing data and regression modeling matches that described previously.^24-26^ To account for missing data, multiple imputation was performed (aregImpute; Hmisc R package in R, Version 4.1.2 [R Project for Statistical Computing]).^27^ We used 20 imputations as described previously.^24^ This uses predictive mean matching with bootstrap draws to build rich additive restricted cubic spline models.^28^ Multiple imputation included all variables listed **Table S1** and **S2** (**SDC**, https://links.lww.com/TP/D333) for deceased and live donors, respectively. Graft/patient survival (cumulative hazard and censoring indicator), DGF, acute rejection and 12-mo eGFR and were included as variables in the multiple imputation model, to preserve associations between variables and outcome.^29,30^

To assess the association of recipient HIV with graft survival, multivariable Cox proportional-hazards regression was performed, pooling results from all 20 imputed datasets, adjusting variance based on both within- and between-imputation variation, using the fit.mult.impute function (Hmisc package in R, Version 4.1.2).^27^ Separate models were built for the deceased and live cohorts and adjusted for a wide range of confounders. Potential confounders were selected based on previous research and clinical expertise; statistical variable selection techniques (eg, stepwise selection) were avoided.^31^

For the main graft survival models, variables with significant right skew were analyzed on the log_2_ scale. As both kidney transplant outcomes and proportion of HIV-positive recipients change over time, it was important to account for era in our models. Transplant year was therefore modeled using a restricted cubic spline. This avoids assumptions of linear associations and offers greater power than splitting into arbitrary ‘eras’. Spline terms had 3 knots in the default positions (10th, 50th, and 90th percentiles).^28^ Additional exploratory models were built with interaction terms to assess whether any impact of recipient HIV has changed over time, or whether the impact of recipient HIV varied depending on recipient ethnicity or IMD. The same model building approach was used for patient survival, DGF and acute rejection (logistic regression), and 12-mo eGFR (multiple linear regression).

Separate models were built, that were identical to the adjusted models described above, except without the inclusion of recipient ethnicity or IMD as confounders. This was done to assess whether previous studies not adjusting for ethnicity and IMD are likely to have overestimated the risk of recipient HIV positivity.

All statistical modeling was performed in the cohorts of first-time transplant recipients. Assessment of retransplants was performed with exploratory crude analyses because of very small number of HIV-positive recipient transplants.

Results from all models are provided as an effect estimate and 95% confidence interval (CI). *P* < 0.05 was considered statistically significant. All analyses were performed in R, Version 4.1.2 (R Project for Statistical Computing)^32^ using the following packages: tidyverse, rms, Hmisc, and survminer.^33–35^

## RESULTS

**FIGURE 1.**
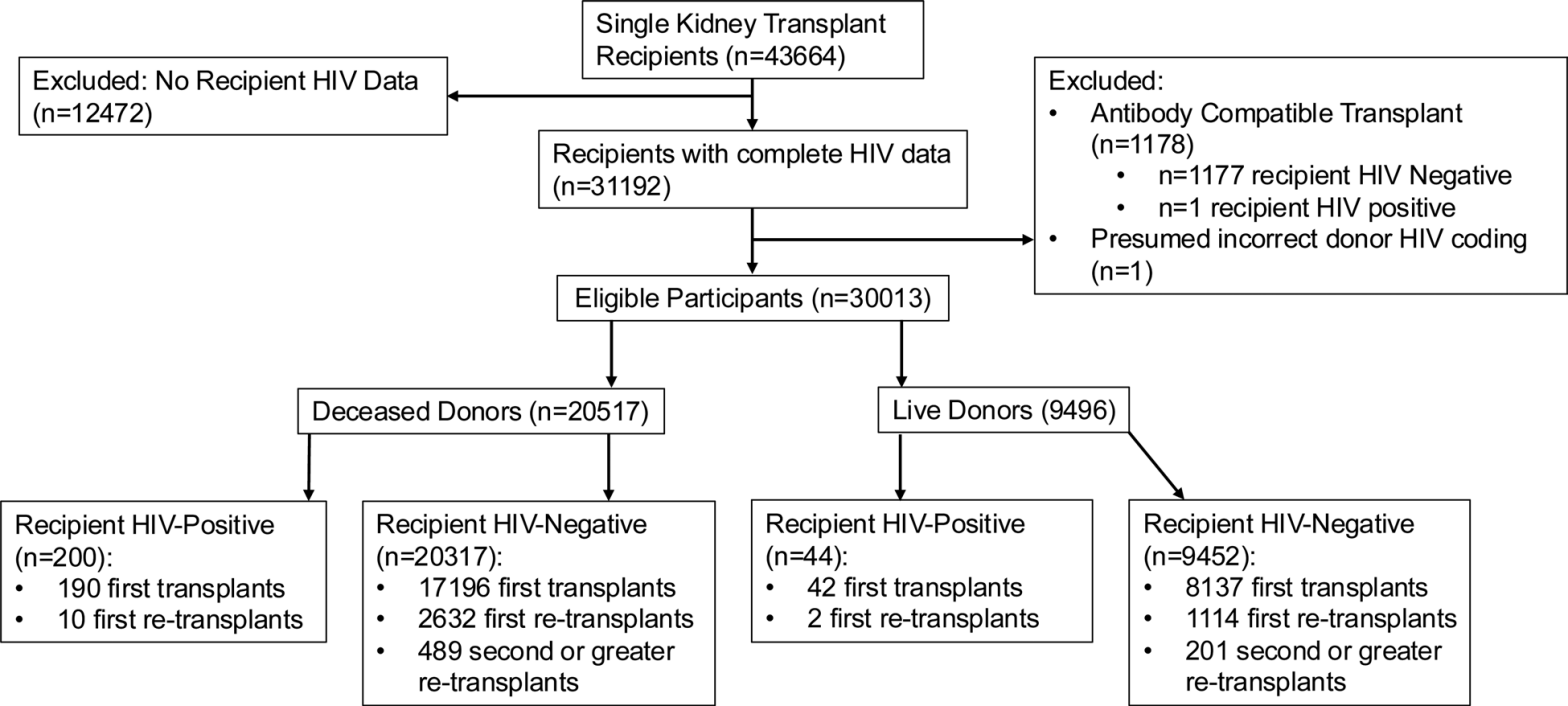
Flow diagram demonstrating inclusion and exclusion criteria and study cohorts.

Figure 1 contains our study flow diagram, number of transplants excluded. We included 30 013 kidney transplant recipients: 20 517 from deceased donors (200 HIV-positive recipients) and 9496 live donors (44 HIV-positive recipients); 17 386 (190 HIV-positive) and 8179 (42 HIV-positive) of these were first-time transplants.

**FIGURE 2.**
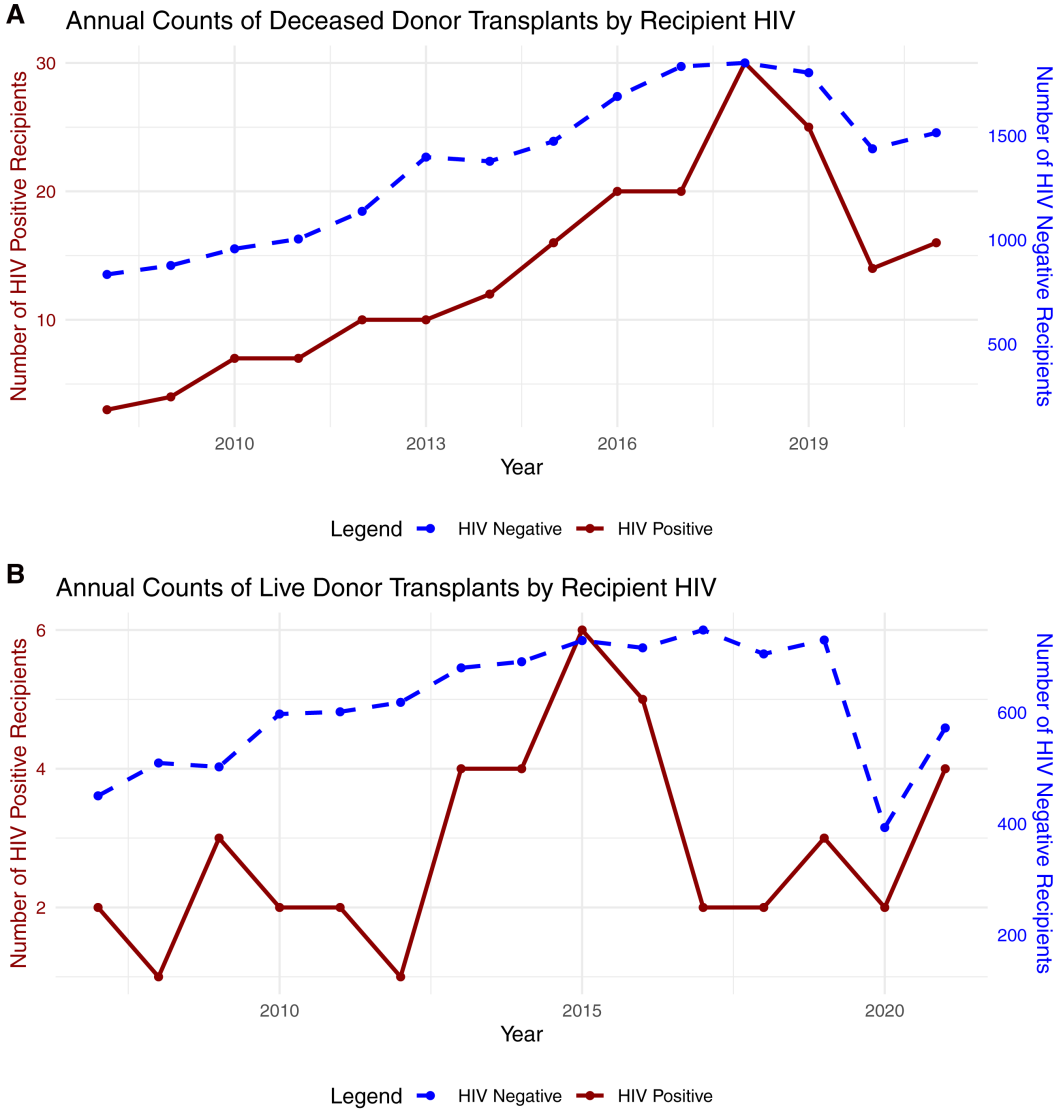
Number of transplants by calendar year and recipient HIV status. Annual counts of deceased (A) and live (B) kidney transplants, stratified by recipient HIV status. Dashed lines and right y-axis represent transplants into HIV-negative recipients. Solid lines and left y-axis represent transplants into HIV-positive recipients.

Key demographics are given in Tables 1 and 2, with full cohort demographics in **Table S1–S4** (**SDC**, https://links.lww.com/TP/D333). The recipients of deceased-donor and live-donor grafts had a median age of 54 y (interquartile range, 43–63 y) and 47 y (interquartile range, 35–58 y), 37% and 39% were female, and 9% and 4% were patients of Black ethnicity, respectively. As shown in Table 1, HIV-positive recipients of deceased-donor grafts were more often of Black ethnicity (73% versus 8%) and had a higher IMD (26.2 versus 16.2). Similar differences were observed for live-donor recipients (Table 2). Overall transplant numbers increased from 2005 to 2019 (Figure 2), with the proportion of HIV-positive recipients increasing over time.

**TABLE 1.** Demographic and clinical characteristics and early posttransplant outcomes in recipients of deceased-donor kidney allografts, excluding retransplants

Characteristic	HIV-negative recipient (N = 17 196)	HIV-positive recipient (N = 190)
Recipient age, y		
Median (Q1–Q3)	54.0 (43.0–63.0)	47.0 (41.3–54.8)
Recipient sex		
Female	6390 (37.2%)	74 (38.9%)
Male	10 804 (62.8%)	116 (61.1%)
Missing	2 (0.0%)	0 (0%)
Recipient ethnicity		
White	12 661 (73.6%)	36 (18.9%)
Black	1380 (8.0%)	138 (72.6%)
Asian	2723 (15.8%)	7 (3.7%)
Other	327 (1.9%)	7 (3.7%)
Missing	105 (0.6%)	2 (1.1%)
Wait time, days		
Median (Q1–Q3)	796 (386–1300)	1260 (627–1790)
Missing	32 (0.2%)	0 (0%)
Recipient IMD		
Median (Q1–Q3)	20.6 (11.3–35.4)	36.4 (22.9–46.0)
Missing	2492 (14.5%)	20 (10.5%)
Donor age, y		
Median (Q1–Q3)	53.0 (41.0–62.0)	50.0 (38.3–58.0)
Donor sex		
Female	7610 (44.3%)	88 (46.3%)
Male	9586 (55.7%)	102 (53.7%)
Delayed graft function		
Immediate function	11 765 (68.4%)	120 (63.2%)
No immediate function	4396 (25.6%)	63 (33.2%)
Missing	1035 (6.0%)	7 (3.7%)
Rejection at 12 mo		
No rejection	10 101 (58.7%)	85 (44.7%)
Rejection	1673 (9.7%)	28 (14.7%)
Missing	5422 (31.5%)	77 (40.5%)
eGFR at 12 mo		
Median (Q1–Q3)	50.4 (35.6–66.7)	42.5 (28.4–58.6)
Missing	1829 (10.6%)	26 (13.7%)

eGFR, estimated glomerular filtration rate; IMD, index of multiple deprivation.

**TABLE 2.** Demographic and clinical characteristics and early posttransplant outcomes in recipients of live-donor kidney allografts, excluding retransplants

Characteristic	HIV-negative recipient (N = 8137)	HIV-positive recipient (N = 42)
Recipient age, y		
Median (Q1–Q3)	47.0 (35.0–58.0)	49.0 (39.0–55.8)
Recipient sex		
Female	3135 (38.5%)	18 (42.9%)
Male	4992 (61.3%)	24 (57.1%)
Missing	10 (0.1%)	0 (0%)
Recipient ethnicity		
White	7030 (86.4%)	19 (45.2%)
Black	278 (3.4%)	21 (50.0%)
Asian	633 (7.8%)	1 (2.4%)
Other	140 (1.7%)	1 (2.4%)
Missing	56 (0.7%)	0 (0%)
Recipient IMD		
Median (Q1–Q3)	16.2 (9.39–28.5)	26.2 (17.1–33.6)
Missing	1482 (18.2%)	6 (14.3%)
Donor age, y		
Median (Q1–Q3)	49.0 (40.0–58.0)	48.5 (39.0–58.0)
Missing	2 (0.0%)	0 (0%)
Donor sex		
Female	4409 (54.2%)	21 (50.0%)
Male	3726 (45.8%)	21 (50.0%)
Missing	2 (0.0%)	0 (0%)
Delayed graft function		
Immediate function	7264 (89.3%)	40 (95.2%)
No immediate function	240 (2.9%)	2 (4.8%)
Missing	633 (7.8%)	0 (0%)
Rejection at 12 mo		
No rejection	5141 (63.2%)	20 (47.6%)
Rejection	932 (11.5%)	12 (28.6%)
Missing	2064 (25.4%)	10 (23.8%)
eGFR at 12 mo		
Median (Q1–Q3)	59.7 (48.2–72.7)	49.2 (40.5–61.9)
Missing	814 (10.0%)	6 (14.3%)

eGFR, estimated glomerular filtration rate; IMD, index of multiple deprivation.

Initial immunosuppression for deceased-donor and live-donor transplants are shown in **Table S5** (**SDC**, https://links.lww.com/TP/D333). Plots of how immunosuppression regimens have changed over time, stratified by recipient HIV status, are given in **Figures S1** and **S2** (**SDC**, https://links.lww.com/TP/D333). Of note, ciclosporin was historically used in preference to tacrolimus in HIV-positive recipients, whereas in recent years tacrolimus is used almost exclusively.

The adjusted comparisons between HIV-positive versus negative recipients for all analyzed outcomes are summarized in Table 3. Splines for changes in outcomes over time are given in **Figures S3** and **S4** (**SDC**, https://links.lww.com/TP/D333).

**TABLE 3.** Summary of all multivariable models

Outcome	HIV-positive vs negative recipient comparison–deceased-donor cohort	HIV-positive vs negative recipient comparison–live donor cohort
Graft survival	aHR = 0.927 (0.609-1.409; *P* = 0.721)	aHR = 1.021 (0.313-3.325; *P* = 0.973)
Patient survival	aHR = 1.275 (0.836-1.945; *P* = 0.259)	aHR = 1.675 (0.815-3.443; *P* = 0.160)
Delayed graft function	aOR = 0.986 (0.699-1.393; *P* = 0.938)	aOR = 1.152 (0.255-5.197; *P* = 0.854)
12-mo rejection	aOR = 1.528 (0.935-2.498; *P* = 0.091)	aOR = 3.430 (1.435-8.194; *P* = 0.006)
12-mo eGFR	–4.150 (–7.388 to –0.912; *P* = 0.012)	–1.949 (–8.357 to 4.458; *P* = 0.551)

Results all represent the outcome in HIV-positive recipients vs HIV-negative recipients adjusted for all variables in the respective multivariable models. Survival was analyzed with Cox regression, delayed graft function and acute rejection with logistic regression, and 12-mo eGFR with linear regression. Full model results, and details of how potential confounders were analyzed, are provided in **Tables S6–S15** (**SDC**, https://links.lww.com/TP/D333). These models include first-time kidney transplants only.

aHR, adjusted hazard ratio; aOR, adjusted odds ratio; eGFR, estimated glomerular filtration rate.

### Impact of Recipient HIV Status on Graft and Patient Survival

**FIGURE 3.**
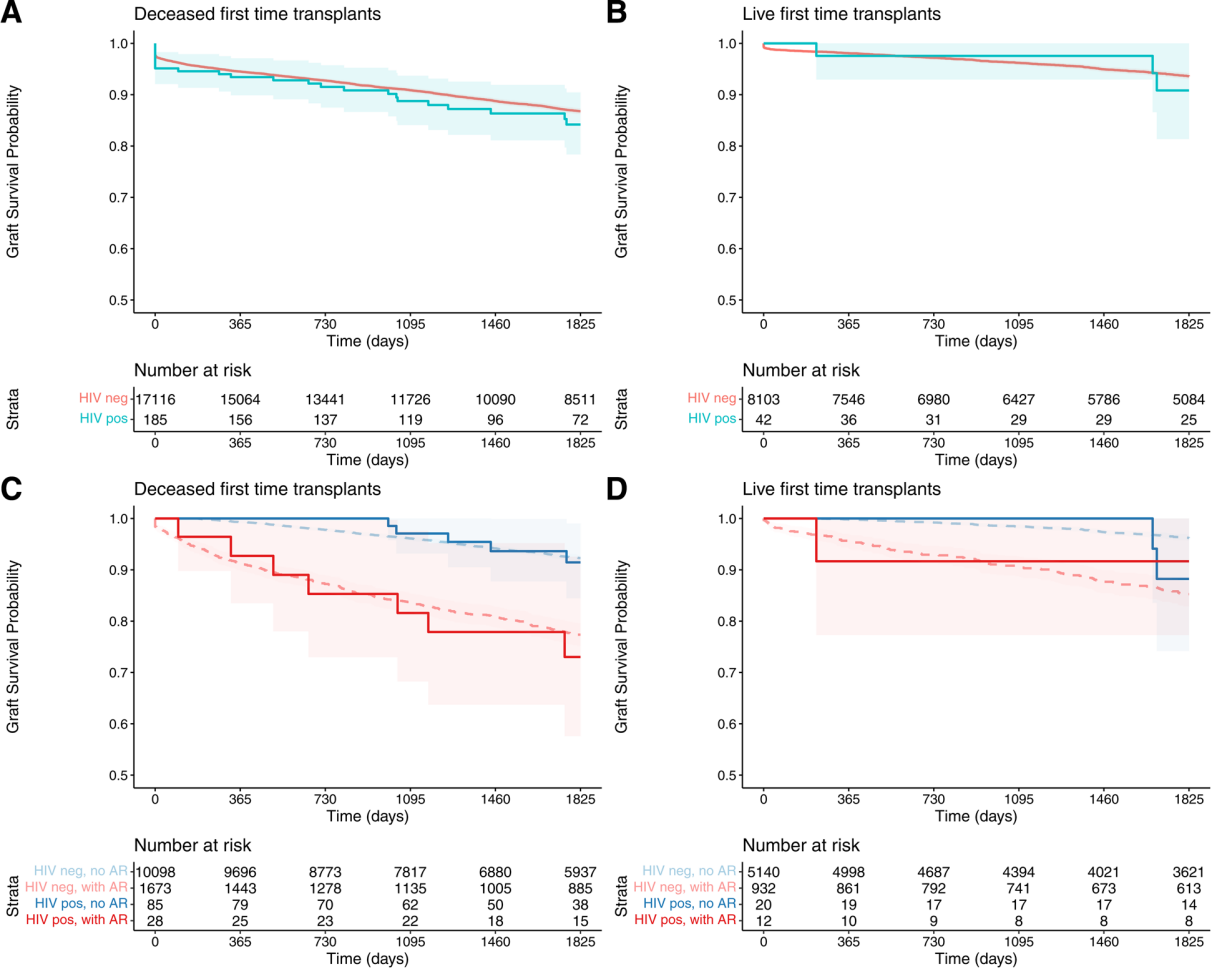
Graft survival following first-time kidney transplantation by recipient HIV status and AR in the first year. Kaplan-Meier plots showing graft survival probability >5 y among first-time kidney transplant recipients. Graft survival stratified by HIV status for recipients of (A) deceased and (B) live donor transplants. Graft survival further stratified by both occurrence of AR and recipient HIV status in (C) deceased-donor and (D) live-donor recipients. Shaded areas indicate 95% confidence intervals. Number at risk is shown beneath each plot. AR, acute rejection.

Figure 3A and B shows Kaplan-Meier graft survival plot up to 5 y posttransplantation for first-time transplants. Five-year graft survival was 86.8% for recipients of deceased-donor grafts and 93.6% for recipients of live-donor grafts. In crude analyses, HIV status showed a nonstatistically significant association with inferior graft survival among recipients of deceased-donor (crude hazard ratio [HR] = 1.34 [95% CI, 0.95-1.89]; *P* = 0.094) and live-donor grafts (crude HR = 1.78 [0.83-3.79]; *P* = 0.137).

After adjustment for all variables in **Tables S6** and **S7** (**SDC**, https://links.lww.com/TP/D333), HIV status was not associated with graft survival in the deceased-donor (adjusted HR [aHR] = 0.93 [0.61-1.41]; *P* = 0.721; **Table S6** [**SDC**, https://links.lww.com/TP/D333]) or live-donor (aHR = 1.02 [0.31-3.33]; *P* = 0.973; **Table S7** [**SDC**, https://links.lww.com/TP/D333]) cohort. These models adjusted for transplant year to account for any era effect, as both outcomes and proportion of HIV-positive transplants changed over time (all results displayed in **Figures S3** and **S4** [**SDC**, https://links.lww.com/TP/D333]). In models adjusting for all factors except recipient ethnicity and IMD, the adjusted HRs for graft loss were higher than in the fully adjusted models, for both the deceased (aHR = 1.10 [0.74-1.66]; *P* = 0.625) and live (aHR = 1.20 [0.38-3.83]; *P* = 0.759) cohorts.

Exploratory models including interaction terms between recipient HIV and transplant year revealed no evidence that the impact of recipient HIV status on graft survival changed over the study period in either the deceased (interaction *P* = 0.154) or live (interaction *P* = 0.614) donor cohort. Further interaction terms provided no evidence that the impact of recipient HIV status differs based on recipient ethnicity or IMD in either the deceased (interaction *P* = 0.527 and *P* = 0.782, respectively) or live (interaction *P* = 0.996 and *P* = 0.891, respectively) cohorts.

Additional exploratory analyses were performed which censored graft survival at 10 y. This adjusted for the same potential confounders and revealed no impact of recipient HIV status on long-term graft survival in either deceased (aHR = 0.97 [0.68-1.39]; *P* = 0.869) or live (aHR = 1.39 [0.60-3.24]; *P* = 0.447) donor cohorts.

Five-year patient survival was 87.1% for recipients of deceased-donor grafts and 94.3% for recipients of live-donor grafts. HIV status was not associated with patient survival in either cohort (summary results in Table 3, with full models in **Tables S8** and **S9** [**SDC**, https://links.lww.com/TP/D333]; deceased aHR = 1.28 [0.84-1.95]; *P* = 0.259 and live aHR = 1.68 [0.82-3.44]; *P* = 0.160). Additional adjusted analyses for patient survival censored at 10 y also revealed no impact of recipient HIV status.

### Impact of Recipient HIV Status on DGF

The proportion of participants experiencing DGF was 33.2% and 25.6% among HIV-positive and HIV-negative recipients of deceased-donor grafts, and 4.8% and 2.9% in recipients of live-donor grafts. In adjusted analyses, recipient HIV status was not associated with DGF in the deceased-donor (adjusted odds ratio [aOR] = 0.99 [0.70-1.39]; *P* = 0.938; **Table S10** [**SDC**, https://links.lww.com/TP/D333]) and live-donor (aOR = 1.15 [0.26-5.20]; *P* = 0.854; **Table S11** [**SDC**, https://links.lww.com/TP/D333]) cohorts.

In models adjusting for all factors in **Tables S10** and **S11** (**SDC**, https://links.lww.com/TP/D333), except for recipient ethnicity and IMD, the impact of HIV status was overestimated in both the deceased-donor (aOR = 1.42 [1.02-1.97]; *P* = 0.039) and live-donor (aOR = 1.30 [0.30-5.77]; *P* = 0.726) cohorts. Exploratory models with interaction terms with year of transplant revealed no evidence that the impact of HIV status differed over time (interaction *P* = 0.209 and *P* = 0.381 for deceased and live cohorts, respectively).

### Impact of Recipient HIV Status on 12-mo Rejection

Crude 12-mo acute rejection rates for HIV-positive versus negative recipients were 24.8% versus 14.2% (deceased) and 37.5% versus 15.3% (live). In crude analyses, HIV status was associated with rejection at 12 mo in both the deceased-donor group (crude OR = 2.01 [1.29-3.15]; *P* = 0.002) and live-donor group (crude OR = 4.02 [1.85-8.74]; *P* < 0.001). In adjusted analyses, HIV-positive recipients showed a trend toward increased acute rejection rate in the deceased-donor cohort (aOR = 1.53 [0.94-2.50]; *P* = 0.091; **Table S12** [**SDC**, https://links.lww.com/TP/D333]). The negative impact of HIV-positive status on acute rejection remained significant in the live-donor cohort (aOR = 3.43 [1.44-8.19]; *P* = 0.006; **Table S13** [**SDC**, https://links.lww.com/TP/D333]).

In models adjusting for all of the same factors, except recipient ethnicity and IMD, the impact of HIV on acute rejection was exaggerated in both the deceased (aOR = 1.98 [1.24-3.17]; *P* = 0.005) and live (aOR = 4.09 [1.77-9.47]; *P* < 0.001) donor cohorts. Exploratory interaction analyses revealed no evidence that the impact of HIV status on acute rejection varied over time (interaction *P* = 0.410 and *P* = 0.905 for deceased and live cohorts, respectively). Kaplan-Meier plots reveal a similar negative impact of acute rejection on graft survival in HIV-positive and HIV-negative recipients (Figure 3C and D; log-rank *P* < 0.001 for both).

### Impact of Recipient HIV Status on 12-mo eGFR

Twelve-month eGFR was 42.5 and 50.4 mL/min/1.73 m^2^ for HIV-positive and HIV-negative recipients of deceased-donor grafts, and 49.2 and 59.7 for live-donor recipients. In crude analyses, HIV status was associated with reduced eGFR in both cohorts: deceased-donor (coefficient, –5.87 [–9.47 to –2.28]; *P* = 0.001) and live-donor (coefficient, –6.94 [–13.60 to –0.27]; *P* = 0.041). After adjustment, the association remained significant in deceased-donor recipients (coefficient, –4.15 [–7.39 to –0.91]; *P* = 0.012; **Table S14** [**SDC**, https://links.lww.com/TP/D333]), but not in live-donor recipients (coefficient, –1.95 [–8.36 to 4.46]; *P* = 0.551; **Table S15** [**SDC**, https://links.lww.com/TP/D333]).

In a model adjusting for all the same factors except ethnicity and IMD, the impact of recipient HIV status on 12-mo eGFR appeared far greater (deceased-donor cohort coefficient,–9.17 [–12.38 to –5.95]; *P* < 0.001 and live-donor cohort coefficient, –4.73 [–11.08 to 1.62]; *P* = 0.144). Exploratory models with interaction terms revealed no evidence that the negative impact of HIV status on eGFR was modified by any of the following: IMD, recipient ethnicity, or year of transplant (interaction *P* all >0.1).

### Graft Survival With Retransplantation in HIV-positive Recipients

Ten HIV-positive recipients underwent retransplantation with a graft from a deceased-donor, all of whom were first-time retransplants. **Figure S5A** and **B** (**SDC**, https://links.lww.com/TP/D333) displays graft survival in retransplants versus first-time transplants. Among HIV-negative recipients of deceased-donor kidneys, Kaplan-Meier estimated 5-y graft survival was 86.8% (95% CI, 86.2%-87.4%) for first-time transplants and 82.6% (95% CI, 81.0%-84.3%) for first retransplants. For HIV-positive recipients, the estimated 5-y graft survival was 84.2% (95% CI, 78.4%-90.5%) for first time and 61.7% (95% CI, 34.9%-100%) for first retransplants.

### Sensitivity Analyses

When excluding those transplanted before 2015 (**Table S16**, **SDC**, https://links.lww.com/TP/D333), or including only participants with complete outcome data for the respective models (**Table S17**, **SDC**, https://links.lww.com/TP/D333), the adjusted impact of recipient HIV status on the various outcomes was in keeping with the full cohort results.

## DISCUSSION

This national population-cohort study demonstrates that, after adjusting for multiple confounding factors, including recipient ethnicity and socioeconomic deprivation, HIV-positive status does not negatively impact death-censored 5-y graft survival, patient survival, or the incidence of DGF. Our findings support the inclusion of HIV-positive individuals on kidney transplantation waiting lists and underscore the need for continued efforts to ensure equitable access to transplantation services.

We have shown that when recipient ethnicity and socioeconomic deprivation are not adjusted for, the risks for HIV-positive recipients were overestimated in our cohort. This underscores the need for caution when interpreting earlier research, which did not adjust for these confounders.^1,6,8,36-38^ Specifically, the largest US studies focusing on the impact of recipient HIV on kidney transplant outcome adjusted for race, but not socioeconomic deprivation.^8,38^ Some US analyses use insurance type (private versus nonprivate) as a surrogate for social deprivation.^39^ However, this binary measure fails to reflect the wide variability in socioeconomic deprivation, which is captured by the continuous IMD scale used here.

In the United Kingdom, Black individuals represent 37% of those diagnosed with HIV in 2023,^40^ and are at sevenfold increased risk of end-stage kidney disease as compared with those of White/other ethnicities.^4^ Despite this dual burden, Black individuals are more likely to experience prolonged waiting times or not be referred for kidney transplantation.^5,7,9,10^ This further highlights the importance of ensuring equitable access to transplantation for HIV-positive recipients, to avoid reinforcing existing racial disparities.

Transplant eligibility for HIV-positive patients is assessed on an individual basis, typically managed by multidisciplinary teams within tertiary centers.^41^ Previously published criteria include a CD4 count generally >200 cells/μL, undetectable plasma HIV RNA on a stable cART regimen, and the absence of recent opportunistic infection or malignancy.^42^ These criteria have remained largely unchanged during the study period. While these thresholds provide a useful framework, clinical judgment remains essential to decision-making.^42^ Following referral, patients undergo a comprehensive review by a multidisciplinary team comprising nephrologists, HIV specialists and transplant surgeons.

Higher rates of acute rejection among HIV-positive kidney transplant recipients have been reported previously. One such study in the US documented biopsy-proven acute rejection rates in HIV-positive recipients of 31% at 1 y and 41% at 3 y, while the National Institutes of Health multicenter study reported a 31% acute rejection at 1 y among HIV-positive recipients, compared with 12.3% in HIV-negative controls.^1,9,37,43-45^

The present study corroborates these findings, demonstrating that this increase in acute rejection risk persists, even with adjustment for ethnicity and socioeconomic deprivation. The underlying causes are complex, multifactorial and not fully understood. Chronic immune activation, immunodeficiency and immune dysregulation in people with HIV likely contribute to this increased risk. Notably, HIV infection depletes activated effector T regulatory cells, which are thought to play a key role in modulating alloreactivity posttransplant.^5,46,47^ Although cytomegalovirus replication has been linked to immune activation in chronic HIV and proposed as a potential contributor to acute rejection,^9^ this could not be explored here given the constraints of the data.

It should be noted that although the adjusted rate of acute rejection was increased, this did not translate into inferior graft survival. Overall, while increased rejection episodes can negatively impact long-term graft survival, the outcome is influenced by the type and timing of rejection, the effectiveness of management strategies, and individual patient and donor factors. This granular data was unfortunately not available for analysis in our study.

In the deceased-donor cohort HIV-positive versus negative recipients had somewhat lower 12-mo eGFR. This reduction in eGFR is likely multifactorial and may not reflect genuine changes in renal function. Many antiretroviral medications inhibit creatinine excretion leading to an artefactual average reduction in eGFR of approximately 8–12 mL/min/1.73m^2^ without reflecting true declines in kidney function.^17^ This complicates accurate comparisons of kidney function in this cohort. While these pharmacological effects are important to consider, our ability to fully interpret their impact is limited by the lack of antiretroviral and immuno-virological data in the UK Transplant Registry.

Our data revealed a 5.4 mL/min/1.73 m^2^ lower eGFR in Black recipients compared with those of White ethnicity, aligning with previously reported ethnic disparities in posttransplant renal function.^5,16,43^ This highlights the importance of adjusting for recipient ethnicity as a confounder, as it is associated with both transplant outcome and recipient HIV status.

An optimal antiretroviral and immunosuppressive regimen for HIV-positive kidney transplant recipients has yet to be defined.^18^ Integrase inhibitors, which lack interactions with transplant-immunosuppression, are increasingly used for the management of HIV in the setting of transplantation. Immunosuppressive protocols are generally similar to those used in HIV-negative recipients,^45,47^ with tacrolimus now preferred over ciclosporin because of its more favorable efficacy profile. Steroid-based induction remains standard practice. However, there is international variation in immunosuppression strategies, with the United States favoring anti-thymocyte globulin and the UK preferring IL2RA,^12,17^ although a recent study indicated that IL2RA are increasingly used in the United States in the setting of HIV-positive recipient transplant.^48^ Notably, Stock et al^1^ reported a high incidence of steroid-resistant acute cellular rejection, although no cases of antibody-mediated acute rejection were observed. Optimizing immunosuppressive strategies in the context of concomitant cART remains a critical area for future research.

These results must be understood within the broader context of immunosuppressive regimens used in the HIV-negative control recipients. As discussed above, the United States may have a higher tendency to use more potent induction agents, especially in high-risk patients.^49^ Furthermore, the United States may have more variability in practice because of a larger number of transplant centers and ongoing clinical trials exploring new immunosuppressive strategies.^50^ The United Kingdom, with its centralized healthcare system, can have more uniform practices across centers.^51^ Such practice variation should be considered when interpreting the generalizability of these results to other settings.

This study is subject to the inherent limitation of registry-based data. Key HIV-specific variables (CD4 count, viral load, antiretroviral regimen, and use of prophylaxis for opportunistic infections) were not collected. We therefore cannot comment on the impact of HIV immune status or antiretroviral regimens on posttransplant outcomes. This makes it difficult to precisely assess the generalizability of our findings to individuals with varying HIV disease control and drug regimens. Furthermore, differences in center or clinician level practices when managing HIV-positive recipients may have introduced bias. This was not assessed in our study and would likely require a prospective trial to evaluate adequately.

Data on posttransplant immunosuppressive regimens were inconsistently recorded, allowing only exploratory analysis on treatment variation. Rejection episodes were registry-reported as treated episodes of acute rejection and will include both biopsy-confirmed and nonbiopsy-confirmed cases. IMD is a small area-level measure (calculated from postcode data) that may not accurately reflect individual-level deprivation or healthcare access; however, it is widely used in epidemiological studies of this nature. Finally, despite the large and representative cohort, residual confounding cannot be excluded.

## CONCLUSIONS

In conclusion, when adjusted for key demographic and clinical factors, HIV-positive recipient status does not adversely affect 5-y graft or patient survival, nor the incidence of DGF in kidney transplant recipients. While higher rates of acute rejection rates and lower 12-mo eGFR were observed, these did not result in poorer overall long-term outcomes. Our analysis also indicates that earlier studies lacking adjustment for ethnicity and socioeconomic deprivation likely exaggerated the risks associated with recipient HIV status, as Black ethnicity and greater deprivation are themselves associated with poorer posttransplant outcomes and are common in HIV-positive cohorts. These findings support the continued inclusion of well-managed individuals with HIV on transplant waiting lists and highlight the need to ensure equitable access to transplantation. Future research should aim to incorporate detailed HIV-specific clinical data and evaluate the benefits of specific immunosuppressive and antiretroviral strategies in this growing patient population.

## ACKNOWLEDGMENTS

The authors thank the National Health Service Blood and Transplant Registry for providing access to the data used in this study and to transplant centers for collecting this data. Also, the authors thank the organ donors and their families for their generosity, donor coordinators, and the wider transplant team who facilitate this work.

## Supplementary Material



## References

[1] Stock PG Barin B Murphy B. Outcomes of kidney transplantation in HIV-infected recipients. N Engl J Med. 2010;363:2004–2014. 21083386 10.1056/NEJMoa1001197 PMC3028983

[2] Sawinski D Murphy B. End-stage renal disease and kidney transplant in HIV-infected patients. Semin Nephrol. 2008;28:581–584. 19013329 10.1016/j.semnephrol.2008.08.002

[3] Kalayjian RC. Renal issues in HIV infection. Curr HIV/AIDS Rep. 2011;8:164–171. 21643783 10.1007/s11904-011-0080-x

[4] Bansi L Hughes A Bhagani S; UK CHIC/ESRF study group. Clinical epidemiology of HIV-associated end-stage renal failure in the UK. AIDS. 2009;23:2517–2521. 19752713 10.1097/QAD.0b013e3283320e12

[5] Hilton R. Human immunodeficiency virus infection and kidney disease. J R Coll Physicians Edinb. 2013;43:236–239; quiz 240. 24087804 10.4997/JRCPE.2013.310

[6] Roland ME Barin B Carlson L. HIV-infected liver and kidney transplant recipients: 1- and 3-year outcomes. Am J Transplant. 2008;8:355–365. 18093266 10.1111/j.1600-6143.2007.02061.x

[7] Gathogo EN Hamzah L Hilton R; UK HIV/Kidney Transplantation Study Group (see appendix). Kidney transplantation in HIV-positive adults: the UK experience. Int J STD AIDS. 2014;25:57–66. 23970634 10.1177/0956462413493266

[8] Locke JE Mehta S Reed RD. A national study of outcomes among HIV-infected kidney transplant recipients. J Am Soc Nephrol. 2015;26:2222–2229. 25791727 10.1681/ASN.2014070726 PMC4552118

[9] Sawinski D Forde KA Eddinger K. Superior outcomes in HIV-positive kidney transplant patients compared to HCV-infected or HIV/HCV co-infected recipients. Kidney Int. 2015;88:341–349. 25807035 10.1038/ki.2015.74 PMC5113138

[10] Muller E Kahn D Mendelson M. Renal transplantation between HIV-positive donors and recipients. N Engl J Med. 2010;362:2336–2337. 20554994 10.1056/NEJMc0900837 PMC5094174

[11] Muller E Barday Z Mendelson M. HIV-positive-to-HIV-positive kidney transplantation—results at 3 to 5 years. N Engl J Med. 2015;372:613–620. 25671253 10.1056/NEJMoa1408896 PMC5090019

[12] Gathogo E Harber M Bhagani S; UK HIV Kidney Transplantation Study Group. Impact of tacrolimus compared with cyclosporin on the incidence of acute allograft rejection in human immunodeficiency virus-positive kidney transplant recipients. Transplantation. 2016;100:871–878. 26413990 10.1097/TP.0000000000000879

[13] Ravanan R Udayaraj U Ansell D. Variation between centres in access to renal transplantation in UK: longitudinal cohort study. BMJ. 2010;341:c3451. 20647283 10.1136/bmj.c3451 PMC2907479

[14] Mupfudze TG Handarova D Noreen SM. Influence of individual- and area-level social determinants of health on likelihood of living versus deceased donor kidney transplantation. Kidney Int Rep. 2025;10:791–802. 40225382 10.1016/j.ekir.2024.12.011 PMC11993228

[15] Stephens MR Evans M Ilham MA. The influence of socioeconomic deprivation on outcomes following renal transplantation in the United Kingdom. Am J Transplant. 2010;10:1605–1612. 20199499 10.1111/j.1600-6143.2010.03041.x

[16] Reese PP Blumberg EA Bloom RD. Kidney transplantation in patients with HIV infection. Adv Chronic Kidney Dis. 2010;17:94–101. 20005493 10.1053/j.ackd.2009.08.004

[17] Yombi JC Pozniak A Boffito M. Antiretrovirals and the kidney in current clinical practice: renal pharmacokinetics, alterations of renal function and renal toxicity. AIDS. 2014;28:621–632. 24983540 10.1097/QAD.0000000000000103

[18] Spencer M Pieri C Hamzah L. Antiretroviral therapy in people with HIV and end-stage kidney disease. AIDS. 2025;39:863–868. 39874132 10.1097/QAD.0000000000004128 PMC12077330

[19] NHS Blood and Transplant. Organ transplantation—How we use your information. Available at https://www.nhsbt.nhs.uk/organ-transplantation/resources/how-we-use-your-information/. Accessed June 12, 2025.

[20] Mahendran B Tingle SJ Malik AK. Racial disparities in outcomes after liver transplantation in the UK: registry analysis. Br J Surg. 2024;111:znae020. 38364060 10.1093/bjs/znae020

[21] Geographic Data Service. Index of multiple deprivation (IMD). Available at https://data.geods.ac.uk/dataset/index-of-multiple-deprivation-imd#. Accessed July 8, 2025.

[22] Stirnadel-Farrant HA Mu G Cooper-Blenkinsopp S. Predictive value of delayed graft function definitions following donation after circulatory death renal transplantation in the United Kingdom. Transpl Res Risk Manag. 2022;14:21–33.

[23] Inker LA Eneanya ND Coresh J; Chronic Kidney Disease Epidemiology Collaboration. New creatinine- and cystatin c–based equations to estimate GFR without race. N Engl J Med. 2021;385:1737–1749. 34554658 10.1056/NEJMoa2102953 PMC8822996

[24] Tingle SJ Chung NDH Malik AK. Donor time to death and kidney transplant outcomes in the setting of a 3-hour minimum wait policy. JAMA Netw Open. 2024;7:e2443353. 39541122 10.1001/jamanetworkopen.2024.43353 PMC11565268

[25] BMJ. Strengthening the Reporting of Observational Studies in Epidemiology (STROBE) statement: guidelines for reporting observational studies. Available at https://www.bmj.com/content/335/7624/806. Accessed April 28, 2025.

[26] STROBE. Strengthening the Reporting of Observational Studies in Epidemiology. Available at https://www.strobe-statement.org/. Accessed June 12, 2025.

[27] Harrell FE Jr. Hmisc: Harrell Miscellaneous. Available at https://CRAN.R-project.org/package=Hmisc. Accessed April 28, 2025.

[28] Harrell FE. Regression Modeling Strategies: With Applications to Linear Models, Logistic and Ordinal Regression, and Survival Analysis. Springer International Publishing; 2015.

[29] Austin PC White IR Lee DS. Missing data in clinical research: a tutorial on multiple imputation. Can J Cardiol. 2021;37:1322. 33276049 10.1016/j.cjca.2020.11.010 PMC8499698

[30] van Ginkel JR Linting M Rippe RCA. Rebutting existing misconceptions about multiple imputation as a method for handling missing data. J Pers Assess. 2020;102:297–308. 30657714 10.1080/00223891.2018.1530680

[31] Heinze G Dunkler D. Five myths about variable selection. Transplant Inter. 2017;30:6–10. 10.1111/tri.12895 27896874

[32] BibSonomy. R: A Language and Environment for Statistical Computing. Available at https://www.bibsonomy.org/bibtex/7469ffee3b07f9167cf47e7555041ee7. Accessed April 28, 2025.

[33] Wickham H Averick M Bryan J. Welcome to the Tidyverse. J Open Source Soft. 2019;4:1686.

[34] Kassambara A Kosinski M Biecek P. survminer: drawing survival curves using ‘ggplot2.’ Available at https://CRAN.R-project.org/package=survminer. Accessed April 28, 2025.

[35] Harrell FE Jr. rms: regression modeling strategies. Available at https://CRAN.R-project.org/package=rms. Accessed April 28, 2025.

[36] Locke JE Montgomery RA Warren DS. Renal transplant in HIV-positive patients: long-term outcomes and risk factors for graft loss. Arch Surg. 2009;144:83–86. 19153330 10.1001/archsurg.2008.508

[37] Zarinsefat A Gulati A Shui A. Long-term outcomes following kidney and liver transplant in recipients with HIV. JAMA Surg. 2022;157:240–247. 34985513 10.1001/jamasurg.2021.6798 PMC8733865

[38] Kucirka LM Durand CM Bae S. Induction immunosuppression and clinical outcomes in kidney transplant recipients infected with human immunodeficiency virus. Am J Transplant. 2016;16:2368–2376. 27111897 10.1111/ajt.13840 PMC4956509

[39] Xia Y Friedmann P Yaffe H. Effect of HCV, HIV and coinfection in kidney transplant recipients: mate kidney analyses. Am J Transplant. 2014;14:2037–2047. 25098499 10.1111/ajt.12847

[40] GOV.UK. HIV: annual data. Available at https://www.gov.uk/government/statistics/hiv-annual-data-tables. Accessed September 22, 2025.

[41] McClure M Singh GJ Rayment M. Clinical outcomes of a combined HIV and renal clinic. Clin Kidney J. 2012;5:530–534. 26069796 10.1093/ckj/sfs141 PMC4400564

[42] Muller E Botha FCJ Barday ZA. Kidney transplantation in HIV positive patients: current practice and management strategies. Transplantation. 2021;105:1492–1501. 33044431 10.1097/TP.0000000000003485 PMC8026768

[43] Malat GE Ranganna KM Sikalas N. High frequency of rejections in HIV-positive recipients of kidney transplantation: a single center prospective trial. Transplantation. 2012;94:1020–1024. 23169224 10.1097/TP.0b013e31826c3947

[44] Locke JE James NT Mannon RB. Immunosuppression regimen and the risk of acute rejection in HIV-infected kidney transplant recipients. Transplantation. 2014;97:446–450. 24162248 10.1097/01.TP.0000436905.54640.8c

[45] NIHR Journals Library. Immunosuppressive therapy for kidney transplantation in adults: a systematic review and economic model. Available at https://www.journalslibrary.nihr.ac.uk/hta/HTA20620. Accessed February 17, 2025.

[46] Booth JW Hamzah L Jose S; HIV/CKD Study and the UK CHIC Study. Clinical characteristics and outcomes of HIV-associated immune complex kidney disease. Nephrol Dial Transplant. 2016;31:2099–2107. 26786550 10.1093/ndt/gfv436

[47] Gilbert J Manji A. Considerations in the management of a kidney transplant patient with HIV. Cureus. 2021;13:e18744. 34659933 10.7759/cureus.18744 PMC8513352

[48] El Rifai R Bhunia K Fontana L. Long-term outcomes of induction immunosuppression for kidney transplant recipients with HIV who have average immunologic risk: an inverse probability treatment weighting analysis. Am J Transplant. 2025;25:756–766. 39515757 10.1016/j.ajt.2024.11.004

[49] Pilch NA Bowman LJ Taber DJ. Immunosuppression trends in solid organ transplantation: the future of individualization, monitoring, and management. Pharmacotherapy. 2021;41:119–131. 33131123 10.1002/phar.2481 PMC8778961

[50] Axelrod DA Naik AS Schnitzler MA. National variation in use of immunosuppression for kidney transplantation: a call for evidence-based regimen selection. Am J Transplant. 2016;16:2453–2462. 26901466 10.1111/ajt.13758 PMC5513703

[51] Jones-Hughes T Snowsill T Haasova M. Immunosuppressive therapy for kidney transplantation in adults: a systematic review and economic model. Health Technol Assess. 2016;20:1–594. 10.3310/hta20620 PMC5018688 27578428

