# Supplementary material for: The Impact of Recipient HIV Status on Kidney Transplant Outcomes in the United Kingdom: Have Previous Studies Overestimated Risk?
Source: Transplantation. 2025 Dec 3;110(3):e670–9. doi: 10.1097/TP.0000000000005585 (PMC12908647; doi:10.1097/TP.0000000000005585)
Supplement: Supplementary file 1 [file tpa-110-e670-s001.pdf]

Supplementary Material

Supplementary Tables

Table S1: Full cohort demographics table for live donors, first time transplants only

	**HIV-Negative Recipient (N=8137)**	**HIV-Positive Recipient (N=42)**	**Overall (N=8179)**
**Recipient Age (yr)**			
Median [Q1,Q3]	47.0 [35.0,58.0]	49.0 [39.0,55.8]	47.0 [35.0,58.0]
**Recipient Sex**			
Female	3135 (38.5%)	18 (42.9%)	3153 (38.6%)
Male	4992 (61.3%)	24 (57.1%)	5016 (61.3%)
Missing	10 (0.1%)	0 (0%)	10 (0.1%)
**Recipient Ethnicity**			
White	7030 (86.4%)	19 (45.2%)	7049 (86.2%)
Black	278 (3.4%)	21 (50.0%)	299 (3.7%)
Asian	633 (7.8%)	1 (2.4%)	634 (7.8%)
Other	140 (1.7%)	1 (2.4%)	141 (1.7%)
Missing	56 (0.7%)	0 (0%)	56 (0.7%)
**Recipient BMI**			
Median [Q1,Q3]	26.1 [23.0,29.6]	24.9 [23.5,30.1]	26.1 [23.0,29.6]
Missing	1594 (19.6%)	12 (28.6%)	1606 (19.6%)
**IMD**			
Median [Q1,Q3]	16.2 [9.39,28.5]	26.2 [17.1,33.6]	16.3 [9.42,28.6]
Missing	1482 (18.2%)	6 (14.3%)	1488 (18.2%)
**Recipient Diabetic Status**			
Recipient Not Diabetic	7513 (92.3%)	37 (88.1%)	7550 (92.3%)
Recipient Diabetic	624 (7.7%)	5 (11.9%)	629 (7.7%)
**Recipient CMV Status**			
CMV Negative	4409 (54.2%)	11 (26.2%)	4420 (54.0%)
CMV Positive	3541 (43.5%)	31 (73.8%)	3572 (43.7%)
Missing	187 (2.3%)	0 (0%)	187 (2.3%)
**Recipient HCV Status**			
HCV Negative	7562 (92.9%)	31 (73.8%)	7593 (92.8%)
HCV Positive	36 (0.4%)	3 (7.1%)	39 (0.5%)
Missing	539 (6.6%)	8 (19.0%)	547 (6.7%)
**Recipient HBV Status**			
HBV Negative	8068 (99.2%)	35 (83.3%)	8103 (99.1%)
HBV Positive	34 (0.4%)	5 (11.9%)	39 (0.5%)
Missing	35 (0.4%)	2 (4.8%)	37 (0.5%)
**Type of Dialysis at Transplant**			
Haemodialysis	3468 (42.6%)	22 (52.4%)	3490 (42.7%)
Peritoneal Dialysis	1495 (18.4%)	10 (23.8%)	1505 (18.4%)
Not on Dialysis	18 (0.2%)	0 (0%)	18 (0.2%)
Missing	3156 (38.8%)	10 (23.8%)	3166 (38.7%)
**Donor Age (yrs)**			
Median [Q1,Q3]	49.0 [40.0,58.0]	48.5 [39.0,58.0]	49.0 [40.0,58.0]
Missing	2 (0.0%)	0 (0%)	2 (0.0%)
**Donor Sex**			
Female	4409 (54.2%)	21 (50.0%)	4430 (54.2%)
Male	3726 (45.8%)	21 (50.0%)	3747 (45.8%)
Missing	2 (0.0%)	0 (0%)	2 (0.0%)
**Donor Ethnicity**			
White	7268 (89.3%)	26 (61.9%)	7294 (89.2%)
Black	214 (2.6%)	13 (31.0%)	227 (2.8%)
Asian	536 (6.6%)	1 (2.4%)	537 (6.6%)

Other	115 (1.4%)	2 (4.8%)	117 (1.4%)
Missing	4 (0.0%)	0 (0%)	4 (0.0%)
**Donor BMI**			
Median [Q1,Q3]	26.4 [23.9,29.0]	25.5 [23.0,27.7]	26.4 [23.9,29.0]
Missing	268 (3.3%)	1 (2.4%)	269 (3.3%)
**HLA Mismatch**			
1	793 (9.7%)	4 (9.5%)	797 (9.7%)
2	1206 (14.8%)	10 (23.8%)	1216 (14.9%)
3	3780 (46.5%)	15 (35.7%)	3795 (46.4%)
4	2312 (28.4%)	13 (31.0%)	2325 (28.4%)
Missing	46 (0.6%)	0 (0%)	46 (0.6%)
**Cold ischaemic time (minutes)**			
Median [Q1,Q3]	208 [146,261]	229 [180,295]	208 [146,261]
Missing	442 (5.4%)	3 (7.1%)	445 (5.4%)
**Primary Renal Disease**			
Glomerulonephritis	1998 (24.6%)	6 (14.3%)	2004 (24.5%)
Cystic Kidney Disease	1220 (15.0%)	4 (9.5%)	1224 (15.0%)
Diabetes	624 (7.7%)	5 (11.9%)	629 (7.7%)
Other	1262 (15.5%)	10 (23.8%)	1272 (15.6%)
Pyelonephritis/Reflux Nephropathy	571 (7.0%)	1 (2.4%)	572 (7.0%)
Renal Vascular Disease	525 (6.5%)	1 (2.4%)	526 (6.4%)
Missing	1937 (23.8%)	15 (35.7%)	1952 (23.9%)
**Highly Sensitised Patient**			
Not Highly Sensitised	7796 (95.8%)	39 (92.9%)	7835 (95.8%)
Highly Sensitised	341 (4.2%)	3 (7.1%)	344 (4.2%)
**Delayed graft function**			
Immediate Function	7264 (89.3%)	40 (95.2%)	7304 (89.3%)
No Immediate Function	240 (2.9%)	2 (4.8%)	242 (3.0%)
Missing	633 (7.8%)	0 (0%)	633 (7.7%)
**Rejection at 3 Months**			
No Rejection	5925 (72.8%)	31 (73.8%)	5956 (72.8%)
Rejection	705 (8.7%)	5 (11.9%)	710 (8.7%)
Missing	1507 (18.5%)	6 (14.3%)	1513 (18.5%)
**Rejection at 12 Months**			
No Rejection	5141 (63.2%)	20 (47.6%)	5161 (63.1%)
Rejection	932 (11.5%)	12 (28.6%)	944 (11.5%)
Missing	2064 (25.4%)	10 (23.8%)	2074 (25.4%)
**eGFR at 12 Months**			
Median [Q1,Q3]	59.7 [48.2,72.7]	49.2 [40.5,61.9]	59.6 [48.2,72.7]
Missing	814 (10.0%)	6 (14.3%)	820 (10.0%)

Table S2: Full cohort demographics table for deceased donors, first time transplants only

	HIV-Negative Recipient (N=17196)	HIV-Positive Recipient (N=190)	Overall (N=17386)
**Recipient Age (yrs)**			
Median [Q1,Q3]	54.0 [43.0,63.0]	47.0 [41.3,54.8]	54.0 [43.0,63.0]
**Recipient Sex**			
Female	6390 (37.2%)	74 (38.9%)	6464 (37.2%)
Male	10804 (62.8%)	116 (61.1%)	10920 (62.8%)
Missing	2 (0.0%)	0 (0%)	2 (0.0%)
**Recipient Ethnicity**			
White	12661 (73.6%)	36 (18.9%)	12697 (73.0%)
Black	1380 (8.0%)	138 (72.6%)	1518 (8.7%)
Asian	2723 (15.8%)	7 (3.7%)	2730 (15.7%)
Other	327 (1.9%)	7 (3.7%)	334 (1.9%)
Missing	105 (0.6%)	2 (1.1%)	107 (0.6%)
**Recipient BMI**			
Median [Q1,Q3]	26.8 [23.6,30.4]	26.2 [22.7,29.5]	26.8 [23.6,30.4]
Missing	3519 (20.5%)	32 (16.8%)	3551 (20.4%)
**Wait-Time**			
Median [Q1,Q3]	796 [386,1300]	1260 [627,1790]	799 [390,1310]
Missing	32 (0.2%)	0 (0%)	32 (0.2%)
**Recipient IMD**			
Median [Q1,Q3]	20.6 [11.3,35.4]	36.4 [22.9,46.0]	20.8 [11.3,35.7]
Missing	2492 (14.5%)	20 (10.5%)	2512 (14.4%)
**Recipient Diabetic Status**			
Recipient Not Diabetic	15164 (88.2%)	182 (95.8%)	15346 (88.3%)
Recipient Diabetic	2032 (11.8%)	8 (4.2%)	2040 (11.7%)
**Recipient CMV Status**			
CMV Negative	7879 (45.8%)	27 (14.2%)	7906 (45.5%)
CMV Positive	8669 (50.4%)	154 (81.1%)	8823 (50.7%)
Missing	648 (3.8%)	9 (4.7%)	657 (3.8%)
**Recipient HCV Status**			
HCV Negative	15662 (91.1%)	155 (81.6%)	15817 (91.0%)
HCV Positive	102 (0.6%)	9 (4.7%)	111 (0.6%)
Missing	1432 (8.3%)	26 (13.7%)	1458 (8.4%)
**Recipient HBV Status**			
HBV Negative	16968 (98.7%)	165 (86.8%)	17133 (98.5%)
HBV Positive	171 (1.0%)	18 (9.5%)	189 (1.1%)
Missing	57 (0.3%)	7 (3.7%)	64 (0.4%)
**Type of Dialysis at Transplant**			
Haemodialysis	10680 (62.1%)	154 (81.1%)	10834 (62.3%)
Peritoneal Dialysis	3886 (22.6%)	23 (12.1%)	3909 (22.5%)
Not on Dialysis	85 (0.5%)	0 (0%)	85 (0.5%)
Missing	2545 (14.8%)	13 (6.8%)	2558 (14.7%)
**Donor Age (yrs)**			
Median [Q1,Q3]	53.0 [41.0,62.0]	50.0 [38.3,58.0]	53.0 [41.0,62.0]
**Donor Sex**			
Female	7610 (44.3%)	88 (46.3%)	7698 (44.3%)
Male	9586 (55.7%)	102 (53.7%)	9688 (55.7%)
**Donor Ethnicity**			
White	16177 (94.1%)	174 (91.6%)	16351 (94.0%)
Black	193 (1.1%)	1 (0.5%)	194 (1.1%)
Asian	402 (2.3%)	7 (3.7%)	409 (2.4%)
Other	288 (1.7%)	4 (2.1%)	292 (1.7%)
Missing	136 (0.8%)	4 (2.1%)	140 (0.8%)
**Donor BMI**			

Median [Q1,Q3]	26.3 [23.3,29.8]	25.7 [22.3,30.1]	26.2 [23.3,29.8]
Missing	170 (1.0%)	0 (0%)	170 (1.0%)
**Donor Type**			
DBD	9817 (57.1%)	125 (65.8%)	9942 (57.2%)
DCD	7008 (40.8%)	58 (30.5%)	7066 (40.6%)
DCD_NRP	371 (2.2%)	7 (3.7%)	378 (2.2%)
**Donor Cardiac Disease History**			
None Previous	14837 (86.3%)	167 (87.9%)	15004 (86.3%)
Known Cardiac Disease	1882 (10.9%)	20 (10.5%)	1902 (10.9%)
Missing	477 (2.8%)	3 (1.6%)	480 (2.8%)
**Donor Diabetes History**			
None Previous	15706 (91.3%)	172 (90.5%)	15878 (91.3%)
Known Diabetic	1225 (7.1%)	15 (7.9%)	1240 (7.1%)
Missing	265 (1.5%)	3 (1.6%)	268 (1.5%)
**Donor Drug Misuse History**			
None Previous	14332 (83.3%)	156 (82.1%)	14488 (83.3%)
Known Drug Use	2355 (13.7%)	32 (16.8%)	2387 (13.7%)
Missing	509 (3.0%)	2 (1.1%)	511 (2.9%)
**Donor Hypertension History**			
None Previous	12079 (70.2%)	140 (73.7%)	12219 (70.3%)
Known Hypertension	4692 (27.3%)	48 (25.3%)	4740 (27.3%)
Missing	425 (2.5%)	2 (1.1%)	427 (2.5%)
**Donor Smoker History**			
None Previous	7600 (44.2%)	78 (41.1%)	7678 (44.2%)
Known Smoking History	9369 (54.5%)	110 (57.9%)	9479 (54.5%)
Missing	227 (1.3%)	2 (1.1%)	229 (1.3%)
**Machine Perfusion**			
No	15733 (91.5%)	180 (94.7%)	15913 (91.5%)
Yes - Hypothermic	821 (4.8%)	3 (1.6%)	824 (4.7%)
Yes - Normothermic	159 (0.9%)	4 (2.1%)	163 (0.9%)
Missing	483 (2.8%)	3 (1.6%)	486 (2.8%)
**HLA Mismatch**			
1	1528 (8.9%)	6 (3.2%)	1534 (8.8%)
2	5185 (30.2%)	53 (27.9%)	5238 (30.1%)
3	8765 (51.0%)	115 (60.5%)	8880 (51.1%)
4	1715 (10.0%)	16 (8.4%)	1731 (10.0%)
Missing	3 (0.0%)	0 (0%)	3 (0.0%)
**Perfusion Quality**			
1	14314 (83.2%)	172 (90.5%)	14486 (83.3%)
2	1035 (6.0%)	8 (4.2%)	1043 (6.0%)
3	234 (1.4%)	4 (2.1%)	238 (1.4%)
4	381 (2.2%)	4 (2.1%)	385 (2.2%)
Missing	1232 (7.2%)	2 (1.1%)	1234 (7.1%)
**Cold Ischaemic Time (mins)**			
Median [Q1,Q3]	822 [635,1040]	788 [611,1020]	822 [635,1040]
Missing	136 (0.8%)	1 (0.5%)	137 (0.8%)
**Donor Creatinine at Retrieval**			
Median [Q1,Q3]	69.0 [54.0,91.0]	64.0 [50.0,87.0]	68.0 [54.0,91.0]
Missing	935 (5.4%)	11 (5.8%)	946 (5.4%)
**Use of Adrenaline at Retrieval**			
No Adrenaline Used	16117 (93.7%)	179 (94.2%)	16296 (93.7%)
Adrenaline Used	1079 (6.3%)	11 (5.8%)	1090 (6.3%)
**Donor Cause of Death**			
ICH	8631 (50.2%)	95 (50.0%)	8726 (50.2%)
Hypoxia	4495 (26.1%)	57 (30.0%)	4552 (26.2%)
Ischaemic Stroke	1204 (7.0%)	8 (4.2%)	1212 (7.0%)
Other	1492 (8.7%)	17 (8.9%)	1509 (8.7%)
Trauma	978 (5.7%)	8 (4.2%)	986 (5.7%)

Missing	396 (2.3%)	5 (2.6%)	401 (2.3%)
**Primary Renal Disease**			
Glomerulonephritis	3491 (20.3%)	49 (25.8%)	3540 (20.4%)
Cystic Kidney Disease	2509 (14.6%)	8 (4.2%)	2517 (14.5%)
Diabetes	2032 (11.8%)	8 (4.2%)	2040 (11.7%)
Other	2505 (14.6%)	60 (31.6%)	2565 (14.8%)
Pyelonephritis/Reflux Nephropathy	982 (5.7%)	6 (3.2%)	988 (5.7%)
Renal Vascular Disease	1439 (8.4%)	16 (8.4%)	1455 (8.4%)
Missing	4238 (24.6%)	43 (22.6%)	4281 (24.6%)
**Highly Sensitised Patient**			
Not Highly Sensitised	16385 (95.3%)	170 (89.5%)	16555 (95.2%)
Highly Sensitised	811 (4.7%)	20 (10.5%)	831 (4.8%)
**Delayed graft function**			
Immediate Function	11765 (68.4%)	120 (63.2%)	11885 (68.4%)
No Immediate Function	4396 (25.6%)	63 (33.2%)	4459 (25.6%)
Missing	1035 (6.0%)	7 (3.7%)	1042 (6.0%)
**Rejection at 3 Months**			
No Rejection	12208 (71.0%)	125 (65.8%)	12333 (70.9%)
Rejection	1199 (7.0%)	17 (8.9%)	1216 (7.0%)
Missing	3789 (22.0%)	48 (25.3%)	3837 (22.1%)
**Rejection at 12 Months**			
No Rejection	10101 (58.7%)	85 (44.7%)	10186 (58.6%)
Rejection	1673 (9.7%)	28 (14.7%)	1701 (9.8%)
Missing	5422 (31.5%)	77 (40.5%)	5499 (31.6%)
**eGFR at 12 Months**			
Median [Q1,Q3]	50.4 [35.6,66.7]	42.5 [28.4,58.6]	50.4 [35.5,66.5]
Missing	1829 (10.6%)	26 (13.7%)	1855 (10.7%)

Table S3: Full cohort demographics table for live donors, including retransplants

Variable	HIV-Negative Recipient (N=9452)	HIV-Positive Recipient (N=44)	Overall (N=9496)
**Recipient Age (yr)**	47.0 [35.0,57.0]	49.0 [39.0,56.5]	47.0 [35.0,57.0]
**Recipient Sex**			
Female	3722 (39.4%)	19 (43.2%)	3741 (39.4%)
Male	5720 (60.5%)	25 (56.8%)	5745 (60.5%)
Missing	10 (0.1%)	0 (0%)	10 (0.1%)
**Recipient Ethnicity**			
White	8214 (86.9%)	20 (45.5%)	8234 (86.7%)
Asian	302 (3.2%)	22 (50.0%)	324 (3.4%)
Black	724 (7.7%)	1 (2.3%)	725 (7.6%)
Other	155 (1.6%)	1 (2.3%)	156 (1.6%)
Missing	57 (0.6%)	0 (0%)	57 (0.6%)
**Recipient BMI**	25.9 [22.9,29.5]	24.7 [23.4,29.8]	25.9 [22.9,29.5]
Missing	1868 (19.8%)	12 (27.3%)	1880 (19.8%)
**Wait-Time**	8.13 [6.89,9.23]	9.33 [7.41,10.1]	8.13 [6.89,9.24]
Missing	2569 (27.2%)	15 (34.1%)	2584 (27.2%)
**Recipient IMD**	4.11 [3.38,4.89]	4.77 [4.19,5.10]	4.11 [3.38,4.89]
Missing	1741 (18.4%)	7 (15.9%)	1748 (18.4%)
**Recipient Diabetic Status**			
Recipient Not Diabetic	8760 (92.7%)	39 (88.6%)	8799 (92.7%)
Recipient Diabetic	692 (7.3%)	5 (11.4%)	697 (7.3%)
**Recipient CMV Status**			
CMV Negative	4960 (52.5%)	13 (29.5%)	4973 (52.4%)
CMV Positive	4269 (45.2%)	31 (70.5%)	4300 (45.3%)
Missing	223 (2.4%)	0 (0%)	223 (2.3%)
**Recipient HCV Status**			
HCV Negative	8778 (92.9%)	32 (72.7%)	8810 (92.8%)
HCV Positive	43 (0.5%)	4 (9.1%)	47 (0.5%)
Missing	631 (6.7%)	8 (18.2%)	639 (6.7%)
**Recipient HBV Status**			
HBV Negative	9375 (99.2%)	36 (81.8%)	9411 (99.1%)
HBV Positive	37 (0.4%)	6 (13.6%)	43 (0.5%)
Missing	40 (0.4%)	2 (4.5%)	42 (0.4%)
**Dialysis at Treatment**			
Haemodialysis	4278 (45.3%)	24 (54.5%)	4302 (45.3%)
Peritoneal Dialysis	1686 (17.8%)	10 (22.7%)	1696 (17.9%)
Not on Dialysis	21 (0.2%)	0 (0%)	21 (0.2%)
Missing	3467 (36.7%)	10 (22.7%)	3477 (36.6%)
**Year of Transplant**	9.50 [6.00,13.0]	10.0 [6.75,12.3]	10.0 [6.00,13.0]
**Number of Kidney Transplants**			
0	8137 (86.1%)	42 (95.5%)	8179 (86.1%)
1	1114 (11.8%)	2 (4.5%)	1116 (11.8%)
Morethan1	201 (2.1%)	0 (0%)	201 (2.1%)
**Donor Age (yrs)**	49.0 [39.0,57.0]	48.5 [37.0,58.3]	49.0 [39.0,57.0]
Missing	3 (0.0%)	0 (0%)	3 (0.0%)
**Donor Sex**			
Female	5065 (53.6%)	23 (52.3%)	5088 (53.6%)
Male	4384 (46.4%)	21 (47.7%)	4405 (46.4%)
Missing	3 (0%)	0 (0%)	3 (0%)
**Donor Ethnicity**			
White	8485 (89.8%)	28 (63.6%)	8513 (89.6%)
Asian	231 (2.4%)	13 (29.5%)	244 (2.6%)
Black	604 (6.4%)	1 (2.3%)	605 (6.4%)
Other	126 (1.3%)	2 (4.5%)	128 (1.3%)

Missing	6 (0.1%)	0 (0%)	6 (0.1%)
**Donor BMI**	26.4 [23.8,29.0]	24.9 [23.0,27.6]	26.4 [23.8,29.0]
Missing	322 (3.4%)	1 (2.3%)	323 (3.4%)
**Donor eGFR**			
Immediate Function	8417 (89.1%)	42 (95.5%)	8459 (89.1%)
No Immediate Function	305 (3.2%)	2 (4.5%)	307 (3.2%)
Missing	730 (7.7%)	0 (0%)	730 (7.7%)
**Rejection at 3 Months**			
No Rejection	6900 (73.0%)	33 (75.0%)	6933 (73.0%)
Rejection	809 (8.6%)	5 (11.4%)	814 (8.6%)
Missing	1743 (18.4%)	6 (13.6%)	1749 (18.4%)
**Rejection at 12 Months**			
No Rejection	5955 (63.0%)	22 (50.0%)	5977 (62.9%)
Rejection	1070 (11.3%)	12 (27.3%)	1082 (11.4%)
Missing	2427 (25.7%)	10 (22.7%)	2437 (25.7%)
**HLA Mismatch**			
1	1016 (10.7%)	4 (9.1%)	1020 (10.7%)
2	1421 (15.0%)	10 (22.7%)	1431 (15.1%)
3	4336 (45.9%)	16 (36.4%)	4352 (45.8%)
4	2629 (27.8%)	14 (31.8%)	2643 (27.8%)
Missing	50 (0.5%)	0 (0%)	50 (0.5%)
**Cold Ischaemic Time (mins)**	7.73 [7.24,8.07]	7.85 [7.50,8.23]	7.73 [7.24,8.07]
Missing	517 (5.5%)	3 (6.8%)	520 (5.5%)
**Primary Renal Disease**			
Glomerulonephritis	2358 (24.9%)	7 (15.9%)	2365 (24.9%)
Cystic Kidney Disease	1297 (13.7%)	4 (9.1%)	1301 (13.7%)
Diabetes	692 (7.3%)	5 (11.4%)	697 (7.3%)
Other	1512 (16.0%)	10 (22.7%)	1522 (16.0%)
Pyelonephritis/Reflux Nephropathy	788 (8.3%)	2 (4.5%)	790 (8.3%)
Renal Vascular Disease	581 (6.1%)	1 (2.3%)	582 (6.1%)
Missing	2224 (23.5%)	15 (34.1%)	2239 (23.6%)
**Highly Sensitised Patient**			
Not Highly Sensitised	8662 (91.6%)	40 (90.9%)	8702 (91.6%)
Highly Sensitised	790 (8.4%)	4 (9.1%)	794 (8.4%)
**eGFR at 12 Months**	60.0 [48.2,73.1]	50.1 [41.7,61.7]	60.0 [48.2,73.1]
Missing	961 (10.2%)	6 (13.6%)	967 (10.2%)

Table S4: Full cohort demographics table for deceased donors, including retransplants

Variable	HIV-Negative Recipient (N=20317)	HIV-Positive Recipient (N=200)	Overall (N=20517)
**Recipient Age (yr)**	53.0 [42.0,62.0]	47.0 [41.0,54.3]	53.0 [42.0,62.0]
**Recipient Sex**			
Female	7594 (37.4%)	78 (39.0%)	7672 (37.4%)
Male	12721 (62.6%)	122 (61.0%)	12843 (62.6%)
Missing	2 (0.0%)	0 (0%)	2 (0.0%)
**Recipient Ethnicity**			
White	15237 (75.0%)	37 (18.5%)	15274 (74.4%)
Asian	1545 (7.6%)	147 (73.5%)	1692 (8.2%)
Black	3065 (15.1%)	7 (3.5%)	3072 (15.0%)
Other	362 (1.8%)	7 (3.5%)	369 (1.8%)
Missing	108 (0.5%)	2 (1.0%)	110 (0.5%)
**Recipient BMI**	26.5 [23.4,30.1]	26.2 [22.8,29.5]	26.5 [23.3,30.1]
Missing	4102 (20.2%)	33 (16.5%)	4135 (20.2%)
**Wait-Time**	9.72 [8.67,10.4]	10.3 [9.30,10.8]	9.72 [8.68,10.4]
Missing	46 (0.2%)	0 (0%)	46 (0.2%)
**Recipient IMD**	4.42 [3.61,5.18]	5.18 [4.53,5.54]	4.43 [3.62,5.18]
Missing	2999 (14.8%)	22 (11.0%)	3021 (14.7%)
**Recipient Diabetic Status**			
Recipient Not Diabetic	18124 (89.2%)	192 (96.0%)	18316 (89.3%)
Recipient Diabetic	2193 (10.8%)	8 (4.0%)	2201 (10.7%)
**Recipient CMV Status**			
CMV Negative	9130 (44.9%)	29 (14.5%)	9159 (44.6%)
CMV Positive	10420 (51.3%)	162 (81.0%)	10582 (51.6%)
Missing	767 (3.8%)	9 (4.5%)	776 (3.8%)
**Recipient HCV Status**			
HCV Negative	18511 (91.1%)	165 (82.5%)	18676 (91.0%)
HCV Positive	127 (0.6%)	9 (4.5%)	136 (0.7%)
Missing	1679 (8.3%)	26 (13.0%)	1705 (8.3%)
**Recipient HBV Status**			
HBV Negative	20058 (98.7%)	174 (87.0%)	20232 (98.6%)
HBV Positive	187 (0.9%)	19 (9.5%)	206 (1.0%)
Missing	72 (0.4%)	7 (3.5%)	79 (0.4%)
**Dialysis at Treatment**			
Haemodialysis	13121 (64.6%)	162 (81.0%)	13283 (64.7%)
Peritoneal Dialysis	4283 (21.1%)	23 (11.5%)	4306 (21.0%)
Not on Dialysis	94 (0.5%)	0 (0%)	94 (0.5%)
Missing	2819 (13.9%)	15 (7.5%)	2834 (13.8%)
**Year of Transplant**	11.0 [7.00,14.0]	12.0 [9.00,14.0]	11.0 [7.00,14.0]
**Number of Kidney Transplants**			
0	17196 (84.6%)	190 (95.0%)	17386 (84.7%)
1	2632 (13.0%)	10 (5.0%)	2642 (12.9%)
Morethan1	489 (2.4%)	0 (0%)	489 (2.4%)
**Donor Age (yrs)**	52.0 [41.0,62.0]	50.0 [38.0,58.0]	52.0 [41.0,62.0]
**Donor Sex**			
Female	9087 (44.7%)	90 (45.0%)	9177 (44.7%)
Male	11230 (55.3%)	110 (55.0%)	11340 (55.3%)
**Donor Ethnicity**			
White	19122 (94.1%)	183 (91.5%)	19305 (94.1%)
Asian	239 (1.2%)	1 (0.5%)	240 (1.2%)
Black	456 (2.2%)	7 (3.5%)	463 (2.3%)
Other	336 (1.7%)	5 (2.5%)	341 (1.7%)
Missing	164 (0.8%)	4 (2.0%)	168 (0.8%)
**Donor BMI**	26.2 [23.3,29.7]	25.7 [22.3,30.0]	26.2 [23.3,29.7]

Missing	198 (1.0%)	0 (0%)	198 (1.0%)
**Donor Type**			
DBD	12063 (59.4%)	134 (67.0%)	12197 (59.4%)
DCD	7819 (38.5%)	59 (29.5%)	7878 (38.4%)
DCD NRP	435 (2.1%)	7 (3.5%)	442 (2.2%)
**Donor eGFR**			
Immediate Function	13722 (67.5%)	126 (63.0%)	13848 (67.5%)
No Immediate Function	5340 (26.3%)	65 (32.5%)	5405 (26.3%)
Missing	1255 (6.2%)	9 (4.5%)	1264 (6.2%)
**Donor Cardiac Disease History**			
None Previous	17589 (86.6%)	175 (87.5%)	17764 (86.6%)
Known Cardiac Disease	2179 (10.7%)	21 (10.5%)	2200 (10.7%)
Missing	549 (2.7%)	4 (2.0%)	553 (2.7%)
**Donor Diabetes History**			
None Previous	18583 (91.5%)	182 (91.0%)	18765 (91.5%)
Known Diabetic	1427 (7.0%)	15 (7.5%)	1442 (7.0%)
Missing	307 (1.5%)	3 (1.5%)	310 (1.5%)
**Donor Drug Misuse History**			
None Previous	16879 (83.1%)	163 (81.5%)	17042 (83.1%)
Known Drug Use	2842 (14.0%)	35 (17.5%)	2877 (14.0%)
Missing	596 (2.9%)	2 (1.0%)	598 (2.9%)
**Donor Hypertension History**			
None Previous	14401 (70.9%)	147 (73.5%)	14548 (70.9%)
Known Hypertension	5437 (26.8%)	50 (25.0%)	5487 (26.7%)
Missing	479 (2.4%)	3 (1.5%)	482 (2.3%)
**Donor Smoker History**			
None Previous	8917 (43.9%)	83 (41.5%)	9000 (43.9%)
Known Smoking History	11141 (54.8%)	115 (57.5%)	11256 (54.9%)
Missing	259 (1.3%)	2 (1.0%)	261 (1.3%)
**Machine Perfusion**			
No	18684 (92.0%)	190 (95.0%)	18874 (92.0%)
Yes - Hypothermic	894 (4.4%)	3 (1.5%)	897 (4.4%)
Yes - Normothermic	191 (0.9%)	4 (2.0%)	195 (1.0%)
Missing	548 (2.7%)	3 (1.5%)	551 (2.7%)
**Rejection at 3 Months**			
No Rejection	14329 (70.5%)	127 (63.5%)	14456 (70.5%)
Rejection	1467 (7.2%)	19 (9.5%)	1486 (7.2%)
Missing	4521 (22.3%)	54 (27.0%)	4575 (22.3%)
**Rejection at 12 Months**			
No Rejection	11781 (58.0%)	86 (43.0%)	11867 (57.8%)
Rejection	2028 (10.0%)	30 (15.0%)	2058 (10.0%)
Missing	6508 (32.0%)	84 (42.0%)	6592 (32.1%)
**HLA Mismatch**			
1	2158 (10.6%)	6 (3.0%)	2164 (10.5%)
2	6135 (30.2%)	55 (27.5%)	6190 (30.2%)
3	10066 (49.5%)	119 (59.5%)	10185 (49.6%)
4	1955 (9.6%)	20 (10.0%)	1975 (9.6%)
Missing	3 (0.0%)	0 (0%)	3 (0.0%)
**Perfusion Quality**			
1	17124 (84.3%)	182 (91.0%)	17306 (84.3%)
2	1176 (5.8%)	8 (4.0%)	1184 (5.8%)
3	258 (1.3%)	4 (2.0%)	262 (1.3%)
4	427 (2.1%)	4 (2.0%)	431 (2.1%)
Missing	1332 (6.6%)	2 (1.0%)	1334 (6.5%)

**Cold Ischaemic Time (mins)**	9.70 [9.34,10.0]	9.63 [9.26,10.0]	9.70 [9.34,10.0]
Missing	158 (0.8%)	1 (0.5%)	159 (0.8%)
**Donor Creatinine at Retrieval**	6.11 [5.78,6.51]	6.04 [5.67,6.49]	6.11 [5.78,6.51]
Missing	1086 (5.3%)	11 (5.5%)	1097 (5.3%)
**Use of Adrenaline at Retrieval**			
No Adrenaline Used	19045 (93.7%)	189 (94.5%)	19234 (93.7%)
Adrenaline Used	1272 (6.3%)	11 (5.5%)	1283 (6.3%)
**Donor Cause of Death**			
ICH	10258 (50.5%)	101 (50.5%)	10359 (50.5%)
Hypoxia	5290 (26.0%)	60 (30.0%)	5350 (26.1%)
Ischaemic Stroke	1410 (6.9%)	8 (4.0%)	1418 (6.9%)
Other	1744 (8.6%)	18 (9.0%)	1762 (8.6%)
Trauma	1142 (5.6%)	8 (4.0%)	1150 (5.6%)
Missing	473 (2.3%)	5 (2.5%)	478 (2.3%)
**Primary Renal Disease**			
Glomerulonephritis	4353 (21.4%)	52 (26.0%)	4405 (21.5%)
Cystic Kidney Disease	2733 (13.5%)	8 (4.0%)	2741 (13.4%)
Diabetes	2193 (10.8%)	8 (4.0%)	2201 (10.7%)
Other	3065 (15.1%)	65 (32.5%)	3130 (15.3%)
Pyelonephritis/Reflux Nephropathy	1402 (6.9%)	6 (3.0%)	1408 (6.9%)
Renal Vascular Disease	1609 (7.9%)	16 (8.0%)	1625 (7.9%)
Missing	4962 (24.4%)	45 (22.5%)	5007 (24.4%)
**Highly Sensitised Patient**			
Not Highly Sensitised	18028 (88.7%)	176 (88.0%)	18204 (88.7%)
Highly Sensitised	2289 (11.3%)	24 (12.0%)	2313 (11.3%)
**eGFR at 12 Months**	50.4 [35.4,66.7]	42.3 [27.8,58.8]	50.3 [35.4,66.6]
Missing	2211 (10.9%)	30 (15.0%)	2241 (10.9%)

Table S5: Induction immunosuppression by recipient HIV status and donor type. Distribution of induction immunosuppressant agent (tacrolimus or ciclosporin) and use of prednisolone at induction among kidney transplant recipients, stratified by recipient HIV status and donor type. Data presented as number (%).

	**HIV-Negative Recipient Deceased-Donor Cohort (N= 20317)**	**HIV-Negative Recipient Live-Donor Cohort (N=9452)**	**HIV-Positive Recipient Deceased-Donor Cohort (N=200)**	**HIV-Positive Recipient Live-Donor Cohort (N=44)**	**Overall (N=30013)**
**Choice of Agent**					
Tacrolimus	19297 (95.0%)	8854 (93.7%)	167 (83.5%)	32 (72.7%)	28350 (94.5%)
Cyclosporin	707 (3.5%)	418 (4.4%)	28 (14.0%)	12 (27.3%)	1165 (3.9%)
Missing	313 (1.5%)	180 (1.9%)	5 (2.5%)	0 (0%)	498 (1.7%)
**Prednisolone at Induction**					
Yes	16881 (83.1%)	8152 (86.2%)	191 (95.5%)	43 (97.7%)	25267 (84.2%)
No	3387 (16.7%)	1211 (12.8%)	8 (4.0%)	1 (2.3%)	4607 (15.4%)
Missing	49 (0.2%)	89 (0.9%)	1 (0.5%)	0 (0%)	139 (0.5%)

Table S6: Multivariable Cox regression model for graft survival (censored at death or 5 years) in the deceased-donor first-time transplants cohort. Variables with right skew were analysed on the Log_2 scale, so effect estimates relate to each time the predictor variable doubles in value. Transplant year was modelled as a restricted cubic spline (results plotted in Figure S3A).

Variable	adjusted HR (95% confidence interval)	P-value
**Recipient HIV Status (Positive)**	0.927 (0.609 to 1.409)	0.721
**Ciclosporin Use**	1.195 (0.956 to 1.495)	0.117
**Recipient Sex (Male)**	0.941 (0.855 to 1.037)	0.219
**Recipient Ethnicity**		
White	Reference	
Asian	0.833 (0.722 to 0.960)	0.012
Black	1.147 (0.976 to 1.350)	0.097
Other	0.665 (0.455 to 0.971)	0.035
**Log_2 Wait-Time**	1.068 (1.032 to 1.106)	<0.001
**Recipient Age, y**	0.981 (0.977 to 0.986)	<0.001
**Recipient BMI**	1.024 (1.014 to 1.034)	<0.001
**Log_2 Recipient Index of Deprivation**	1.081 (1.026 to 1.139)	0.004
**Recipient CMV Status (Positive)**	1.039 (0.940 to 1.149)	0.450
**Recipient HCV Status (Positive)**	1.208 (0.727 to 2.008)	0.465
**Dialysis at Treatment**		
Haemodialysis	Reference	
Peritoneal Dialysis	0.836 (0.747 to 0.936)	0.002
Not on Dialysis	0.510 (0.186 to 1.402)	0.192
**Donor Type**		
DBD	Reference	
DCD without NRP	1.274 (1.140 to 1.424)	<0.001
DCD with NRP	0.975 (0.610 to 1.558)	0.915
**Donor Sex (Male)**	0.987 (0.895 to 1.089)	0.798
**Donor Age, y**	1.019 (1.015 to 1.023)	<0.001
**Donor BMI**	0.996 (0.987 to 1.005)	0.342
**Donor Ethnicity**		
White	Reference	
Asian	0.993 (0.735 to 1.341)	0.962
Black	0.936 (0.617 to 1.421)	0.757
Other	0.953 (0.649 to 1.398)	0.804
**Donor Past Cardiac Disease**	1.080 (0.938 to 1.243)	0.283
**Donor Past Diabetes**	1.146 (0.971 to 1.353)	0.107
**Donor Past Drug Abuse**	0.810 (0.683 to 0.960)	0.015
**Donor Past Hypertension**	1.308 (1.177 to 1.454)	<0.001
**Donor Past Smoker**	1.064 (0.968 to 1.168)	0.199
**Machine Perfusion**		
No	Reference	
Hypothermic	0.912 (0.741 to 1.123)	0.387
Normothermic	0.686 (0.332 to 1.416)	0.308
**NHSBT HLA Mismatch Level**		
1	Reference	
2	1.055 (0.869 to 1.281)	0.588
3	1.293 (1.068 to 1.564)	0.008
4	1.258 (0.993 to 1.594)	0.057
**Quality of Cold Perfusion**		
Good	Reference	
Fair	1.161 (0.970 to 1.389)	0.103
Poor	1.434 (1.027 to 2.003)	0.034
Patchy	1.235 (0.923 to 1.652)	0.156
**Log_2 Cold Ischaemic Time**	1.145 (1.046 to 1.252)	0.003
**Log_2 Donor Creatinine at Retrieval**	1.210 (1.121 to 1.307)	<0.001

**Donor Adrenaline (Yes)**	0.990 (0.819 to 1.198)	0.921
**Log_2 Length of Hospital Stay**	1.063 (1.009 to 1.120)	0.021
**Donor Cause of Death**		
Intracerebral Haemorrhage	Reference	
Hypoxia	0.766 (0.675 to 0.869)	<0.001
Ischaemic Stroke	0.926 (0.776 to 1.106)	0.397
Other	0.989 (0.838 to 1.169)	0.900
Trauma	0.895 (0.724 to 1.106)	0.304
**Primary Renal Disease**		
Glomerulonephritis	Reference	
Cystic Kidney Disease	0.664 (0.523 to 0.842)	<0.001
Diabetes	1.001 (0.865 to 1.160)	0.984
Other	0.964 (0.829 to 1.122)	0.640
Pyelonephritis/Reflux Nephropathy	0.992 (0.812 to 1.212)	0.937
Renal Vascular Disease	0.945 (0.792 to 1.127)	0.528
**Highly Sensitised Patient (CRF > 85%)**	1.045 (0.840 to 1.301)	0.692
**Overall p-Value for Transplant Year Spline**	RCS terms	0.043

Table S7: Multivariable Cox regression model for graft survival (censored at death or 5 years) in the live-donor first-time transplants cohort. Variables with right skew were analysed on the Log_2 scale, so effect estimates relate to each time the predictor variable doubles in value. Transplant year was modelled as a restricted cubic spline (results plotted in Figure S4A).

Variable	Adjusted Hazard Ratio (95% confidence interval)	P-value
**Recipient HIV Status (Positive)**	1.021 (0.313 to 3.325)	0.973
**Ciclosporin Use**	0.832 (0.545 to 1.271)	0.395
**Recipient Sex (Male)**	0.737 (0.606 to 0.895)	0.002
**Recipient Ethnicity**		
White	Reference	
Asian	0.727 (0.411 to 1.288)	0.275
Black	1.666 (0.956 to 2.904)	0.072
Other	0.647 (0.274 to 1.532)	0.323
**Log_2 Wait-Time**	1.025 (0.964 to 1.089)	0.431
**Recipient Age, y**	0.979 (0.972 to 0.987)	<0.001
**Recipient BMI**	1.010 (0.986 to 1.034)	0.406
**Log_2 Recipient Index of Deprivation**	1.200 (1.079 to 1.335)	<0.001
**Recipient CMV Status (Positive)**	0.946 (0.772 to 1.161)	0.596
**Recipient HCV Status (Positive)**	0.694 (0.103 to 4.662)	0.707
**Dialysis at Treatment**		
Haemodialysis	Reference	
Peritoneal Dialysis	0.867 (0.685 to 1.097)	0.235
Not on Dialysis	0.954 (0.125 to 7.254)	0.963
**Donor Sex (Male)**	0.839 (0.693 to 1.015)	0.070
**Donor Age, y**	1.016 (1.007 to 1.024)	<0.001
**Donor BMI**	1.013 (0.987 to 1.039)	0.333
**Donor Ethnicity**		
White	Reference	
Asian	1.378 (0.767 to 2.478)	0.283
Black	1.123 (0.591 to 2.136)	0.723
Other	1.761 (0.860 to 3.609)	0.122
**NHSBT HLA Mismatch Level**		
1	Reference	
2	1.460 (0.943 to 2.259)	0.089
3	1.659 (1.124 to 2.449)	0.011
4	1.717 (1.141 to 2.584)	0.010
**Log_2 Cold Ischaemic Time**	1.162 (1.025 to 1.317)	0.019
**Primary Renal Disease**		
Glomerulonephritis	Reference	
Cystic Kidney Disease	0.686 (0.495 to 0.950)	0.023
Diabetes	0.812 (0.539 to 1.224)	0.320
Other	0.963 (0.730 to 1.270)	0.790
Pyelonephritis/Reflux Nephropathy	0.657 (0.437 to 0.988)	0.044
Renal Vascular Disease	0.915 (0.616 to 1.358)	0.659
**Highly Sensitised Patient (CRF > 85%)**	0.866 (0.534 to 1.403)	0.559
**Overall p-Value for Transplant Year Spline**	RCS terms	<0.001

Table S8: Multivariable Cox regression model for patient survival in the deceased-donor first-time transplants cohort. Variables with right skew were analysed on the Log_2 scale, so effect estimates relate to each time the predictor variable doubles in value. Transplant year was modelled as a restricted cubic spline (results plotted in Figure S3B).

Variable	Adjusted HR (95% confidence interval)	P-value
**Recipient HIV Status (Positive)**	1.275 (0.836 to 1.945)	0.259
**Ciclosporin Use**	1.098 (0.938 to 1.284)	0.244
**Recipient Sex (Male)**	1.082 (1.000 to 1.171)	0.049
**Recipient Ethnicity**		
White	Reference	
Asian	0.807 (0.724 to 0.900)	<0.001
Black	0.704 (0.602 to 0.823)	<0.001
Other	0.545 (0.385 to 0.773)	<0.001
**Log_2 Wait-Time**	1.055 (1.021 to 1.090)	0.001
**Recipient Age, y**	1.064 (1.060 to 1.068)	<0.001
**Recipient BMI**	1.002 (0.992 to 1.011)	0.744
**Log_2 Recipient Index of Deprivation**	1.129 (1.087 to 1.173)	<0.001
**Recipient CMV Status (Positive)**	1.028 (0.951 to 1.112)	0.488
**Recipient HCV Status (Positive)**	1.086 (0.692 to 1.706)	0.719
**Dialysis at Treatment**		
Haemodialysis	Reference	
Peritoneal Dialysis	0.775 (0.707 to 0.850)	<0.001
Not on Dialysis	0.880 (0.522 to 1.484)	0.633
**Donor Type**		
DBD	Reference	
DCD without NRP	1.042 (0.951 to 1.142)	0.375
DCD with NRP	0.873 (0.576 to 1.323)	0.522
**Donor Sex (Male)**	0.943 (0.875 to 1.016)	0.125
**Donor Age, y**	1.006 (1.003 to 1.009)	<0.001
**Donor BMI**	0.998 (0.991 to 1.005)	0.533
**Donor Ethnicity**		
White	Reference	
Asian	1.154 (0.898 to 1.483)	0.262
Black	0.904 (0.620 to 1.317)	0.598
Other	1.299 (0.971 to 1.739)	0.078
**Donor Past Cardiac Disease**	1.080 (0.971 to 1.202)	0.158
**Donor Past Diabetes**	1.030 (0.905 to 1.173)	0.653
**Donor Past Drug Abuse**	0.985 (0.857 to 1.133)	0.837
**Donor Past Hypertension**	1.099 (1.014 to 1.191)	0.022
**Donor Past Smoker**	0.999 (0.929 to 1.073)	0.968
**Machine Perfusion**		
No	Reference	
Hypothermic	0.975 (0.850 to 1.118)	0.719
Normothermic	0.879 (0.481 to 1.607)	0.676
**NHSBT HLA Mismatch Level**		
1	Reference	
2	1.056 (0.918 to 1.214)	0.446
3	1.136 (0.995 to 1.296)	0.059
4	1.188 (1.008 to 1.400)	0.040
**Quality of Cold Perfusion**		
Good	Reference	
Fair	1.167 (1.015 to 1.341)	0.030
Poor	0.988 (0.734 to 1.328)	0.934
Patchy	0.938 (0.746 to 1.179)	0.582
**Log_2 Cold Ischaemic Time**	1.140 (1.064 to 1.221)	<0.001
**Log_2 Donor Creatinine at Retrieval**	1.006 (0.947 to 1.070)	0.836
**Donor Adrenaline (Yes)**	0.948 (0.818 to 1.099)	0.480

**Log_2 Length of Hospital Stay**	0.996 (0.958 to 1.036)	0.850
**Donor Cause of Death**		
Intracerebral Haemorrhage	Reference	
Hypoxia	1.051 (0.954 to 1.157)	0.314
Ischaemic Stroke	1.079 (0.945 to 1.232)	0.264
Other	0.992 (0.873 to 1.128)	0.904
Trauma	0.919 (0.781 to 1.082)	0.311
**Primary Renal Disease**		
Glomerulonephritis	Reference	
Cystic Kidney Disease	0.841 (0.737 to 0.960)	0.010
Diabetes	1.808 (1.290 to 2.536)	<0.001
Other	1.118 (0.973 to 1.284)	0.116
Pyelonephritis/Reflux Nephropathy	1.156 (0.967 to 1.383)	0.111
Renal Vascular Disease	1.166 (1.008 to 1.349)	0.039
**Highly Sensitised Patient (CRF > 85%)**	1.019 (0.850 to 1.223)	0.836
**Overall p-Value for Transplant Year Spline**	RCS terms	0.012

Table S9: Multivariable Cox regression model for patient survival in the live-donor first-time transplants cohort. Variables with right skew were analysed on the Log_2 scale, so effect estimates relate to each time the predictor variable doubles in value. Transplant year was modelled as a restricted cubic spline (results plotted in Figure S4B).

**Variable**	**Adjusted Hazard Ratio (95% confidence interval)**	**P-value**
**Recipient HIV Status (Positive)**	1.675 (0.815 to 3.443)	0.160
**Ciclosporin Use**	1.093 (0.857 to 1.394)	0.473
**Recipient Sex (Male)**	1.110 (0.965 to 1.277)	0.145
**Recipient Ethnicity**		
White	Reference	
Asian	0.929 (0.631 to 1.369)	0.710
Black	0.993 (0.603 to 1.635)	0.978
Other	1.065 (0.555 to 2.044)	0.851
**Log_2 Wait-Time**	1.037 (0.996 to 1.080)	0.078
**Recipient Age, y**	1.061 (1.055 to 1.067)	<0.001
**Recipient BMI**	0.994 (0.977 to 1.012)	0.515
**Log_2 Recipient Index of Deprivation**	1.111 (1.033 to 1.194)	0.004
**Recipient CMV Status (Positive)**	1.054 (0.919 to 1.210)	0.453
**Recipient HCV Status (Positive)**	1.550 (0.790 to 3.042)	0.202
**Dialysis at Treatment**		
Haemodialysis	Reference	
Peritoneal Dialysis	0.770 (0.654 to 0.907)	0.002
Not on Dialysis	1.300 (0.319 to 5.293)	0.714
**Donor Sex (Male)**	0.969 (0.847 to 1.109)	0.647
**Donor Age, y**	1.007 (1.002 to 1.013)	0.010
**Donor BMI**	0.997 (0.980 to 1.015)	0.780
**Donor Ethnicity**		
White	Reference	
Asian	0.866 (0.568 to 1.322)	0.506
Black	0.843 (0.474 to 1.499)	0.561
Other	0.628 (0.272 to 1.452)	0.277
**NHSBT HLA Mismatch Level**		
1	Reference	
2	1.188 (0.887 to 1.592)	0.248
3	1.297 (1.009 to 1.667)	0.043
4	1.110 (0.855 to 1.441)	0.432
**Log_2 Cold Ischaemic Time**	1.044 (0.958 to 1.136)	0.328
**Primary Renal Disease**		
Glomerulonephritis	Reference	
Cystic Kidney Disease	1.073 (0.819 to 1.406)	0.609
Diabetes	2.859 (2.259 to 3.619)	<0.001
Other	1.319 (0.992 to 1.753)	0.057
Pyelonephritis/Reflux Nephropathy	1.180 (0.879 to 1.584)	0.270
Renal Vascular Disease	1.228 (0.922 to 1.636)	0.160
**Highly Sensitised Patient (CRF > 85%)**	0.849 (0.611 to 1.180)	0.330
**Overall p-Value for Transplant Year Spline**	RCS terms	0.697

Table S10: Multivariable logistic regression model for delayed graft function in the deceased-donor first-time transplants cohort. Variables with right skew were analysed on the Log_2 scale, so effect estimates relate to each time the predictor variable doubles in value. Transplant year was modelled as a restricted cubic spline (results plotted in Figure S3C).

**Variable**	**Adjusted odds ratio (95% confidence interval)**	**P-value**
**Recipient HIV Status (Positive)**	0.986 (0.699 to 1.393)	0.938
**Ciclosporin Use**	1.357 (1.124 to 1.639)	0.001
**Recipient Sex (Male)**	1.285 (1.183 to 1.397)	<0.001
**Recipient Ethnicity**		
White	Reference	
Asian	1.094 (0.971 to 1.233)	0.138
Black	1.745 (1.518 to 2.006)	<0.001
Other	0.846 (0.640 to 1.120)	0.243
**Log_2 Wait-Time**	1.188 (1.153 to 1.224)	<0.001
**Recipient Age, y**	0.994 (0.990 to 0.998)	0.003
**Recipient BMI**	1.045 (1.035 to 1.054)	<0.001
**Log_2 Recipient Index of Deprivation**	1.019 (0.978 to 1.063)	0.368
**Recipient CMV Status (Positive)**	1.064 (0.978 to 1.157)	0.149
**Recipient HCV Status (Positive)**	1.463 (0.929 to 2.303)	0.101
**Dialysis at Treatment**		
Haemodialysis	Reference	
Peritoneal Dialysis	0.402 (0.363 to 0.446)	<0.001
Not on Dialysis	0.475 (0.260 to 0.865)	0.015
**Donor Type**		
DBD	Reference	
DCD without NRP	2.698 (2.457 to 2.964)	<0.001
DCD with NRP	1.660 (1.198 to 2.300)	0.002
**Donor Sex (Male)**	1.077 (0.991 to 1.169)	0.080
**Donor Age, y**	1.017 (1.013 to 1.020)	<0.001
**Donor BMI**	1.014 (1.006 to 1.021)	<0.001
**Donor Ethnicity**		
White	Reference	
Asian	0.992 (0.771 to 1.275)	0.948
Black	0.623 (0.431 to 0.901)	0.012
Other	0.839 (0.618 to 1.141)	0.263
**Donor Past Cardiac Disease**	1.027 (0.911 to 1.159)	0.661
**Donor Past Diabetes**	1.127 (0.975 to 1.304)	0.105
**Donor Past Drug Abuse**	0.820 (0.722 to 0.931)	0.002
**Donor Past Hypertension**	1.191 (1.083 to 1.309)	<0.001
**Donor Past Smoker**	1.056 (0.976 to 1.143)	0.177
**Machine Perfusion**		
No	Reference	
Hypothermic	0.690 (0.579 to 0.823)	<0.001
Normothermic	0.734 (0.452 to 1.190)	0.210
**NHSBT HLA Mismatch Level**		
1	Reference	
2	1.157 (0.975 to 1.374)	0.095
3	1.204 (1.019 to 1.422)	0.029
4	1.271 (1.043 to 1.548)	0.017
**Quality of Cold Perfusion**		
Good	Reference	
Fair	1.338 (1.160 to 1.544)	<0.001
Poor	1.423 (1.068 to 1.896)	0.016
Patchy	1.417 (1.115 to 1.801)	0.004
**Log_2 Cold Ischaemic Time**	1.349 (1.250 to 1.456)	<0.001
**Log_2 Donor Creatinine at Retrieval**	1.473 (1.381 to 1.571)	<0.001

**Donor Adrenaline (Yes)**	0.997 (0.850 to 1.170)	0.974
**Log_2 Length of Hospital Stay**	1.187 (1.136 to 1.241)	<0.001
**Donor Cause of Death**		
Intracerebral Haemorrhage	Reference	
Hypoxia	0.933 (0.845 to 1.030)	0.170
Ischaemic Stroke	1.075 (0.926 to 1.247)	0.343
Other	0.995 (0.862 to 1.149)	0.949
Trauma	0.765 (0.640 to 0.915)	0.003
**Primary Renal Disease**		
Glomerulonephritis	Reference	
Cystic Kidney Disease	0.859 (0.744 to 0.991)	0.037
Diabetes	1.098 (0.772 to 1.561)	0.603
Other	0.920 (0.807 to 1.050)	0.216
Pyelonephritis/Reflux Nephropathy	0.857 (0.708 to 1.037)	0.112
Renal Vascular Disease	0.884 (0.758 to 1.030)	0.115
**Highly Sensitised Patient (CRF > 85%)**	1.221 (1.015 to 1.469)	0.034
**Overall p-Value for Transplant Year Spline**	RCS terms	<0.001

Table S11: Multivariable logistic regression model for delayed graft function in the live-donor first-time transplants cohort. Variables with right skew were analysed on the Log_2 scale, so effect estimates relate to each time the predictor variable doubles in value. Transplant year was modelled as a restricted cubic spline (results plotted in Figure S4C).

Variable	Adjusted Odds Ratio (95% Confidence Interval)	P-value
**Recipient HIV Status (Positive)**	1.152 (0.255 to 5.197)	0.854
**Ciclosporin Use**	0.601 (0.322 to 1.121)	0.110
**Recipient Sex (Male)**	1.267 (0.954 to 1.682)	0.102
**Recipient Ethnicity**		
White	Reference	
Asian	1.348 (0.688 to 2.644)	0.384
Black	2.625 (1.293 to 5.332)	0.008
Other	2.511 (1.074 to 5.869)	0.034
**Log_2 Wait-Time**	1.155 (1.051 to 1.268)	0.003
**Recipient Age, y**	0.992 (0.981 to 1.002)	0.116
**Recipient BMI**	1.049 (1.017 to 1.082)	0.003
**Log_2 Recipient Index of Deprivation**	0.970 (0.842 to 1.117)	0.670
**Recipient CMV Status (Positive)**	1.210 (0.913 to 1.605)	0.184
**Recipient HCV Status (Positive)**	1.343 (0.289 to 6.238)	0.706
**Dialysis at Treatment**		
Haemodialysis	Reference	
Peritoneal Dialysis	0.499 (0.347 to 0.717)	<0.001
Not on Dialysis	1.107 (0.147 to 8.340)	0.922
**Donor Sex (Male)**	1.079 (0.827 to 1.407)	0.575
**Donor Age, y**	1.030 (1.018 to 1.042)	<0.001
**Donor BMI**	1.017 (0.980 to 1.056)	0.367
**Donor Ethnicity**		
White	Reference	
Asian	0.922 (0.444 to 1.916)	0.828
Black	0.556 (0.225 to 1.374)	0.204
Other	0.367 (0.083 to 1.614)	0.185
**NHSBT HLA Mismatch Level**		
1	Reference	
2	0.466 (0.265 to 0.820)	0.008
3	0.770 (0.503 to 1.178)	0.228
4	0.723 (0.457 to 1.144)	0.166
**Log_2 Cold Ischaemic Time**	1.275 (1.059 to 1.534)	0.010
**Primary Renal Disease**		
Glomerulonephritis	Reference	
Cystic Kidney Disease	0.862 (0.591 to 1.258)	0.442
Diabetes	0.760 (0.432 to 1.338)	0.341
Other	1.171 (0.783 to 1.751)	0.443
Pyelonephritis/Reflux Nephropathy	1.295 (0.793 to 2.113)	0.301
Renal Vascular Disease	0.972 (0.561 to 1.686)	0.920
**Highly Sensitised Patient (CRF > 85%)**	0.856 (0.437 to 1.676)	0.651
**Overall p-Value for Transplant Year Spline**	RCS terms	<0.001

Table S12: Multivariable logistic regression model for occurrence of 12-month acute rejection in the deceased-donor first-time transplants cohort. Variables with right skew were analysed on the Log_2 scale, so effect estimates relate to each time the predictor variable doubles in value. Transplant year was modelled as a restricted cubic spline (results plotted in Figure S3D).

**Variable**	**Adjusted odds ratio (95% confidence interval)**	**P-value**
**Recipient HIV Status (Positive)**	1.528 (0.935 to 2.498)	0.091
**Ciclosporin Use**	1.640 (1.322 to 2.034)	<0.001
**Recipient Sex (Male)**	1.250 (1.120 to 1.394)	<0.001
**Recipient Ethnicity**		
White	Reference	
Asian	0.764 (0.653 to 0.893)	<0.001
Black	1.277 (1.068 to 1.525)	0.007
Other	1.128 (0.766 to 1.661)	0.542
**Log_2 Wait-Time**	1.023 (0.985 to 1.062)	0.245
**Recipient Age, y**	0.976 (0.971 to 0.981)	<0.001
**Recipient BMI**	1.029 (1.016 to 1.041)	<0.001
**Log_2 Recipient Index of Deprivation**	0.951 (0.902 to 1.002)	0.061
**Recipient CMV Status (Positive)**	1.113 (0.991 to 1.250)	0.070
**Recipient HCV Status (Positive)**	1.481 (0.795 to 2.759)	0.216
**Dialysis at Treatment**		
Haemodialysis	Reference	
Peritoneal Dialysis	0.955 (0.845 to 1.078)	0.457
Not on Dialysis	1.123 (0.548 to 2.301)	0.751
**Donor Type**		
DBD	Reference	
DCD without NRP	1.148 (1.016 to 1.297)	0.027
DCD with NRP	1.849 (1.212 to 2.819)	0.004
**Donor Sex (Male)**	0.979 (0.879 to 1.090)	0.697
**Donor Age, y**	1.015 (1.010 to 1.021)	<0.001
**Donor BMI**	0.999 (0.988 to 1.009)	0.804
**Donor Ethnicity**		
White	Reference	
Asian	1.358 (0.977 to 1.888)	0.069
Black	1.197 (0.782 to 1.831)	0.408
Other	1.231 (0.838 to 1.808)	0.290
**Donor Past Cardiac Disease**	0.834 (0.698 to 0.998)	0.047
**Donor Past Diabetes**	0.823 (0.673 to 1.005)	0.056
**Donor Past Drug Abuse**	0.845 (0.702 to 1.017)	0.074
**Donor Past Hypertension**	1.101 (0.977 to 1.242)	0.116
**Donor Past Smoker**	0.978 (0.881 to 1.086)	0.678
**Machine Perfusion**		
No	Reference	
Hypothermic	0.597 (0.458 to 0.779)	<0.001
Normothermic	0.371 (0.173 to 0.798)	0.011
**NHSBT HLA Mismatch Level**		
1	Reference	
2	0.927 (0.748 to 1.150)	0.491
3	1.307 (1.056 to 1.618)	0.014
4	1.398 (1.070 to 1.826)	0.014
**Quality of Cold Perfusion**		
Good	Reference	
Fair	1.036 (0.833 to 1.289)	0.748
Poor	1.524 (1.050 to 2.213)	0.027
Patchy	1.380 (1.002 to 1.902)	0.049
**Log_2 Cold Ischaemic Time**	0.922 (0.834 to 1.020)	0.114
**Log_2 Donor Creatinine at Retrieval**	1.135 (1.044 to 1.234)	0.003
**Donor Adrenaline (Yes)**	1.145 (0.918 to 1.428)	0.228

**Log_2 Length of Hospital Stay**	1.058 (1.001 to 1.119)	0.046
**Donor Cause of Death**		
Intracerebral Haemorrhage	Reference	
Hypoxia	0.811 (0.703 to 0.935)	0.004
Ischaemic Stroke	0.992 (0.814 to 1.209)	0.936
Other	0.980 (0.801 to 1.198)	0.841
Trauma	0.873 (0.694 to 1.097)	0.243
**Primary Renal Disease**		
Glomerulonephritis	Reference	
Cystic Kidney Disease	0.979 (0.829 to 1.157)	0.805
Diabetes	1.058 (0.887 to 1.262)	0.532
Other	1.062 (0.898 to 1.256)	0.484
Pyelonephritis/Reflux Nephropathy	1.174 (0.943 to 1.461)	0.152
Renal Vascular Disease	1.001 (0.807 to 1.241)	0.996
**Highly Sensitised Patient (CRF > 85%)**	1.511 (1.181 to 1.935)	0.001
**Overall p-Value for Transplant Year Spline**	RCS terms	<0.001

Table S13: Multivariable logistic regression model for 12-month acute rejection in the live-donor first-time transplants cohort. Variables with right skew were analysed on the Log_2 scale, so effect estimates relate to each time the predictor variable doubles in value. Transplant year was modelled as a restricted cubic spline (results plotted in Figure S4D).

Variable	Adjusted Odds Ratio (95% Confidence Interval)	P-value
**Recipient HIV Status (Positive)**	3.430 (1.435 to 8.194)	0.006
**Ciclosporin Use**	1.847 (1.408 to 2.424)	<0.001
**Recipient Sex (Male)**	1.172 (1.009 to 1.360)	0.037
**Recipient Ethnicity**		
White	Reference	
Asian	0.766 (0.468 to 1.252)	0.287
Black	1.202 (0.737 to 1.962)	0.461
Other	1.337 (0.737 to 2.426)	0.339
**Log_2 Wait-Time**	1.017 (0.961 to 1.076)	0.560
**Recipient Age, y**	0.980 (0.974 to 0.985)	<0.001
**Recipient BMI**	1.026 (1.009 to 1.042)	0.002
**Log_2 Recipient Index of Deprivation**	0.969 (0.889 to 1.055)	0.462
**Recipient CMV Status (Positive)**	1.317 (1.140 to 1.520)	<0.001
**Recipient HCV Status (Positive)**	1.579 (0.677 to 3.680)	0.290
**Dialysis at Treatment**		
Haemodialysis	Reference	
Peritoneal Dialysis	0.961 (0.774 to 1.193)	0.719
Not on Dialysis	1.457 (0.443 to 4.786)	0.535
**Donor Sex (Male)**	0.881 (0.766 to 1.013)	0.076
**Donor Age, y**	1.014 (1.008 to 1.020)	<0.001
**Donor BMI**	0.987 (0.967 to 1.007)	0.200
**Donor Ethnicity**		
White	Reference	
Asian	0.745 (0.448 to 1.240)	0.257
Black	0.934 (0.533 to 1.638)	0.811
Other	1.062 (0.558 to 2.020)	0.854
**NHSBT HLA Mismatch Level**		
1	Reference	
2	1.428 (0.993 to 2.054)	0.055
3	2.411 (1.755 to 3.314)	<0.001
4	3.222 (2.317 to 4.481)	<0.001
**Log_2 Cold Ischaemic Time**	1.044 (0.958 to 1.138)	0.325
**Primary Renal Disease**		
Glomerulonephritis	Reference	
Cystic Kidney Disease	0.901 (0.727 to 1.117)	0.341
Diabetes	0.709 (0.521 to 0.967)	0.030
Other	0.989 (0.799 to 1.225)	0.922
Pyelonephritis/Reflux Nephropathy	1.029 (0.786 to 1.347)	0.834
Renal Vascular Disease	0.906 (0.665 to 1.234)	0.531
**Highly Sensitised Patient (CRF > 85%)**	0.997 (0.679 to 1.464)	0.988
**Overall p-Value for Transplant Year Spline**	RCS terms	<0.001

Table S14: Multivariable linear regression model for recipient 12-Month eGFR in the deceased-donor first-time transplants cohort. Results show adjusted change in 12-month eGFR. Variables with right skew were analysed on the Log_2 scale, so effect estimates relate to each time the predictor variable doubles in value. Transplant year was modelled as a restricted cubic spline (results plotted in Figure S3E).

**Variable**	**Adjusted change (95% confidence interval)**	**P-value**
**Recipient HIV Status (Positive)**	-4.150 (-7.388 to -0.912)	0.012
**Ciclosporin Use**	-3.002 (-4.726 to -1.278)	<0.001
**Recipient Sex (Male)**	1.959 (1.280 to 2.639)	<0.001
**Recipient Ethnicity**		
White	Reference	
Asian	5.875 (4.895 to 6.855)	<0.001
Black	-5.315 (-6.587 to -4.043)	<0.001
Other	4.263 (1.944 to 6.582)	<0.001
**Log_2 Wait-Time**	-0.268 (-0.503 to -0.034)	0.025
**Recipient Age (years)**	-0.054 (-0.083 to -0.024)	<0.001
**Recipient BMI**	-0.503 (-0.584 to -0.421)	<0.001
**Log_2 Recipient Index of Deprivation**	-0.416 (-0.773 to -0.059)	0.022
**Recipient CMV Status (Positive)**	-0.235 (-0.925 to 0.456)	0.506
**Recipient HCV Status (Positive)**	-3.392 (-7.175 to 0.392)	0.079
**Dialysis at Treatment**		
Haemodialysis	Reference	
Peritoneal Dialysis	0.078 (-0.734 to 0.890)	0.850
Not on Dialysis	2.598 (-2.564 to 7.760)	0.324
**Donor Type**		
DBD	Reference	
DCD without NRP	-4.360 (-5.141 to -3.579)	<0.001
DCD with NRP	1.766 (-0.978 to 4.509)	0.207
**Donor Sex (Male)**	2.842 (2.151 to 3.533)	<0.001
**Donor Age (years)**	-0.605 (-0.631 to -0.579)	<0.001
**Donor BMI**	0.148 (0.087 to 0.209)	<0.001
**Donor Ethnicity**		
White	Reference	
Asian	-3.351 (-5.438 to -1.264)	0.002
Black	-1.455 (-4.614 to 1.703)	0.366
Other	1.055 (-1.430 to 3.540)	0.406
**Donor Past Cardiac Disease**	-0.922 (-1.976 to 0.133)	0.087
**Donor Past Diabetes**	-2.386 (-3.599 to -1.173)	<0.001
**Donor Past Drug Abuse**	3.403 (2.350 to 4.455)	<0.001
**Donor Past Hypertension**	-4.067 (-4.855 to -3.279)	<0.001
**Donor Past Smoker**	0.351 (-0.311 to 1.012)	0.299
**Machine Perfusion**		
No	Reference	
Hypothermic	1.435 (-0.032 to 2.901)	0.055
Normothermic	-1.649 (-5.715 to 2.418)	0.427
**NHSBT HLA Mismatch Level**		
1	Reference	
2	-0.239 (-1.501 to 1.022)	0.710
3	-0.886 (-2.131 to 0.359)	0.163
4	-0.172 (-1.746 to 1.402)	0.830
**Quality of Cold Perfusion**		
Good	Reference	
Fair	-2.916 (-4.281 to -1.551)	<0.001
Poor	-5.694 (-8.396 to -2.993)	<0.001
Patchy	-3.881 (-5.920 to -1.842)	<0.001
**Log_2 Cold Ischaemic Time**	-1.551 (-2.142 to -0.959)	<0.001
**Log_2 Donor Creatinine at Retrieval**	-3.411 (-3.949 to -2.872)	<0.001

**Donor Adrenaline (Yes)**	0.534 (-0.779 to 1.848)	0.425
**Log_2 Length of Hospital Stay**	-1.375 (-1.746 to -1.005)	<0.001
**Donor Cause of Death**		
Intracerebral Haemorrhage	Reference	
Hypoxia	3.489 (2.607 to 4.372)	<0.001
Ischaemic Stroke	0.801 (-0.476 to 2.078)	0.219
Other	1.399 (0.174 to 2.624)	0.025
Trauma	3.334 (1.941 to 4.728)	<0.001
**Primary Renal Disease**		
Glomerulonephritis	Reference	
Cystic Kidney Disease	1.356 (0.127 to 2.585)	0.031
Diabetes	1.197 (-0.036 to 2.431)	0.057
Other	-0.296 (-1.370 to 0.777)	0.588
Pyelonephritis/Reflux Nephropathy	-1.859 (-3.462 to -0.257)	0.023
Renal Vascular Disease	1.329 (-0.161 to 2.819)	0.080
**Highly Sensitised Patient (CRF > 85%)**	-1.474 (-2.996 to 0.048)	0.058
**Overall P-value for Transplant Year Spline**	RCS terms	<0.001

Table S15: Multivariable linear regression model for recipient 12-month eGFR in the live-donor first-time transplants cohort. Results show adjusted change in 12-month eGFR. Variables with right skew were analysed on the Log_2 scale, so effect estimates relate to each time the predictor variable doubles in value. Transplant year was modelled as a restricted cubic spline (results plotted in Figure S4E).

Variable	Adjusted change (95% confidence interval)	P-value
**Recipient HIV Status (Positive)**	-1.949 (-8.357 to 4.458)	0.551
**Cyclosporin Use**	-2.366 (-4.414 to -0.318)	0.024
**Recipient Sex (Male)**	2.012 (1.111 to 2.913)	<0.001
**Recipient Ethnicity**		
White	Reference	
Asian	6.930 (4.356 to 9.504)	<0.001
Black	-6.986 (-10.236 to -3.735)	<0.001
Other	1.663 (-1.901 to 5.228)	0.360
**Log_2 Wait-Time**	-0.175 (-0.437 to 0.088)	0.192
**Recipient Age (years)**	-0.096 (-0.129 to -0.063)	<0.001
**Recipient BMI**	-0.436 (-0.540 to -0.332)	<0.001
**Log_2 Recipient Index of Deprivation**	-0.451 (-0.944 to 0.042)	0.073
**Recipient CMV Status (Positive)**	-0.590 (-1.510 to 0.330)	0.208
**Recipient HCV Status (Positive)**	5.315 (-1.153 to 11.782)	0.107
**Dialysis at Treatment**		
Haemodialysis	Reference	
Peritoneal Dialysis	0.119 (-1.055 to 1.292)	0.843
Not on Dialysis	-3.071 (-11.185 to 5.044)	0.458
**Donor Sex (Male)**	3.139 (2.296 to 3.982)	<0.001
**Donor Age (years)**	-0.576 (-0.612 to -0.540)	<0.001
**Donor BMI**	0.116 (0.001 to 0.231)	0.048
**Donor Ethnicity**		
White	Reference	
Asian	-3.637 (-6.408 to -0.867)	0.010
Black	-3.491 (-7.014 to 0.033)	0.052
Other	-1.378 (-5.252 to 2.497)	0.486
**NHSBT HLA Mismatch Level**		
1	Reference	
2	-1.182 (-2.883 to 0.519)	0.173
3	-1.368 (-2.822 to 0.086)	0.065
4	-1.035 (-2.587 to 0.517)	0.191
**Log_2 Cold Ischaemic Time**	-1.489 (-2.055 to -0.924)	<0.001
**Primary Renal Disease**		
Glomerulonephritis	Reference	
Cystic Kidney Disease	0.532 (-0.843 to 1.906)	0.449
Diabetes	3.672 (1.873 to 5.471)	<0.001
Other	-1.690 (-3.133 to -0.247)	0.022
Pyelonephritis/Reflux Nephropathy	-1.782 (-3.468 to -0.096)	0.038
Renal Vascular Disease	1.433 (-0.482 to 3.348)	0.142
**Highly Sensitised Patient (CRF > 85%)**	0.953 (-1.135 to 3.041)	0.371
**Overall p-Value for Transplant Year Spline**	RCS terms	<0.001

Table S16 - Sensitivity analyses including only patients transplanted from 2015 onwards. Results all represent the outcome in HIV-positive recipients versus HIV-negative recipients adjusted for all variables in the respective multivariable models.

**Outcome**	**HIV-Positive versus Negative Recipient Comparison - Deceased Donor Cohort**	**HIV-Positive versus Negative Recipient Comparison - Live Donor Cohort**
**Graft survival**	aHR=0.770 (0.442 to 1.339) P=0.354	aHR=1.646 (0.372 to 7.286) P=0.511
**Patient survival**	aHR=1.079 (0.610 to 1.906) P=0.795	aHR=1.820 (0.631 to 5.247) P=0.268
**Delayed Graft Function**	aOR=0.988 (0.650 to 1.502) P=0.956	aOR=2.426 (0.471 to 12.507) P=0.289
**12-month rejection**	aOR=1.477 (0.727 to 3.001) P=0.280	aOR=3.189 (0.756 to 13.450) P=0.114
**12-month eGFR**	-4.167 (-8.145 to -0.190), P=0.040	-2.663 (-11.286 to 5.960), P=0.545

Table S17 - Sensitivity analyses including only patients with complete data for the respective outcome variable in each model (otherwise approach identical to main models, including multiple imputation for covariates). Results all represent the outcome in HIV-positive recipients versus HIV-negative recipients adjusted for all variables in the respective multivariable models.

**Outcome**	**HIV-Positive versus Negative Recipient Comparison - Deceased Donor Cohort**	**HIV-Positive versus Negative Recipient Comparison - Live Donor Cohort**
**Graft survival**	aHR=0.927 (0.609 to 1.409) P=0.721	aHR=1.021 (0.313 to 3.325) P=0.973
**Patient survival**	aHR=1.275 (0.836 to 1.945) P=0.259	aHR=1.675 (0.815 to 3.443) P=0.160
**Delayed Graft Function**	aOR=0.989 (0.699 to 1.400) P=0.950	aOR=1.041 (0.230 to 4.701) P=0.959
**12-month rejection**	aOR=1.381 (0.869 to 2.197) P=0.172	aOR=2.621 (1.189 to 5.779) P=0.017
**12-month eGFR**	-4.366 (-7.542 to -1.190), P=0.007	-2.593 (-8.715 to 3.528), P=0.406

Supplementary Figures

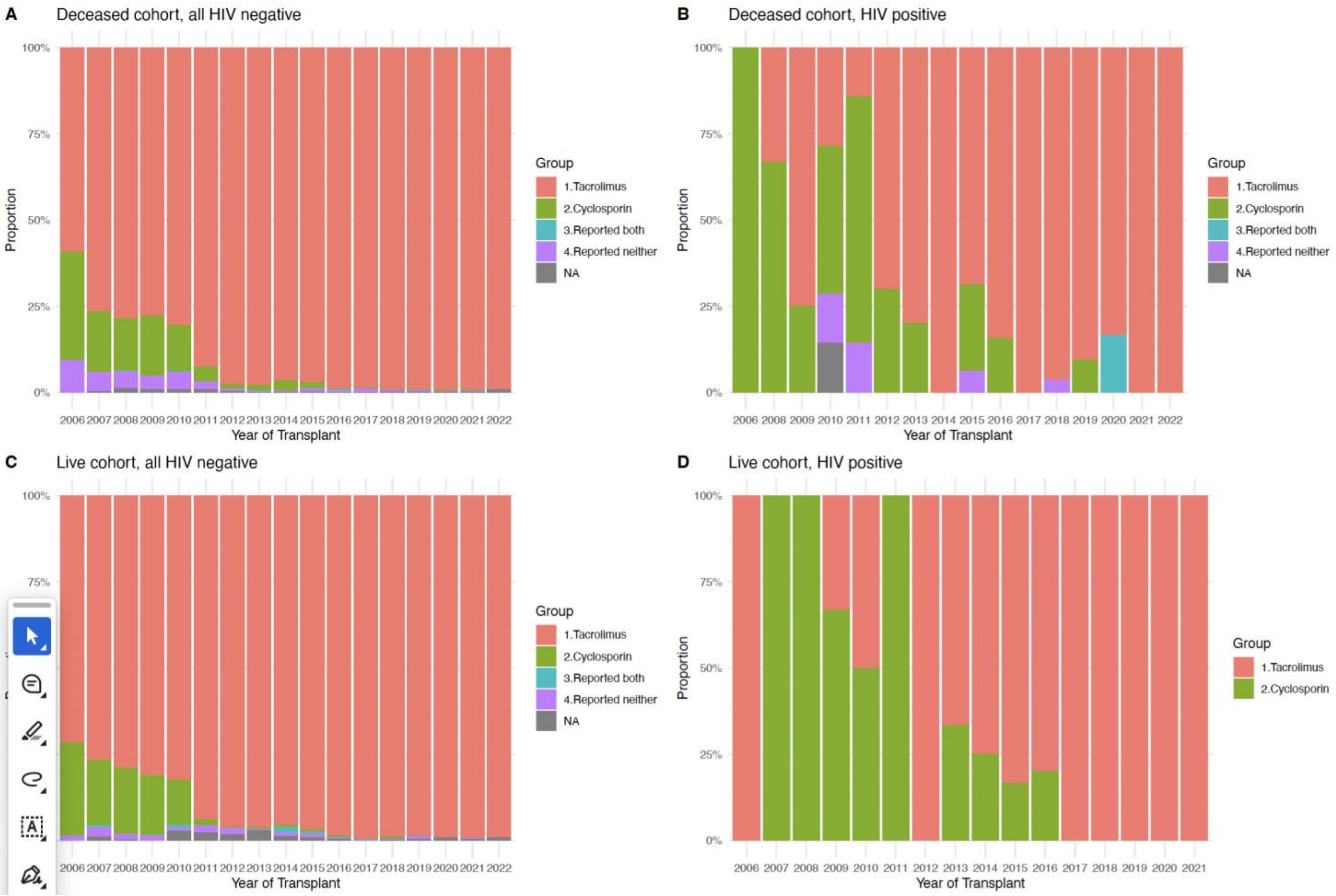

Figure S1: Plots showing tacrolimus versus ciclosporin at time of transplant across transplant year, stratified by HIV status and donor cohort: (A) HIV-negative, deceased cohort; (B) HIV-positive, deceased cohort; (C) HIV-negative, live cohort; (D) HIV-positive, live cohort.

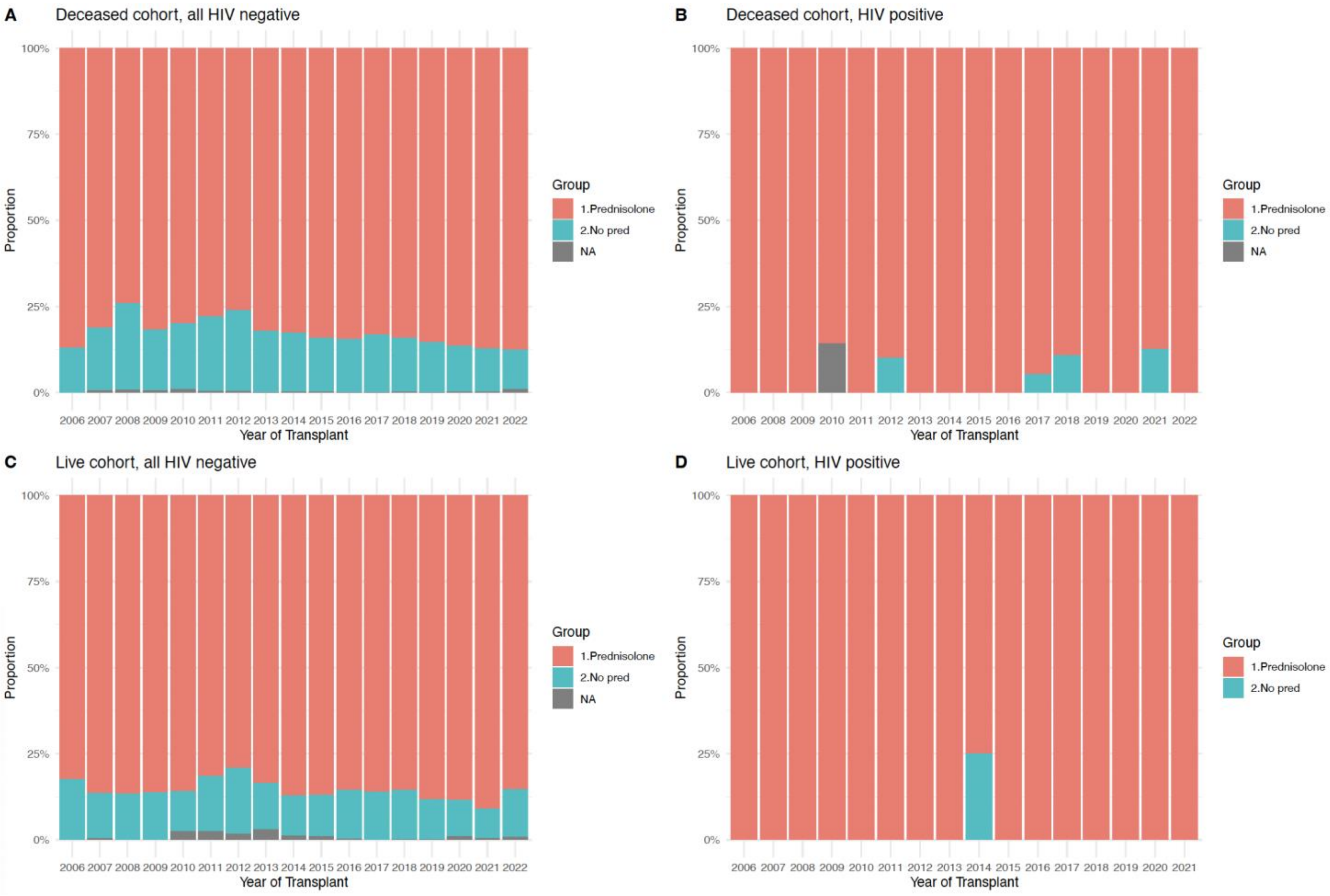

Figure S2: Plots of prednisolone count at time of transplant across transplant year stratified by HIV status and donor cohort: (A) HIV-negative, deceased cohort; (B) HIV-positive, deceased cohort; (C) HIV-negative, live cohort; (D) HIV-positive, live cohort.

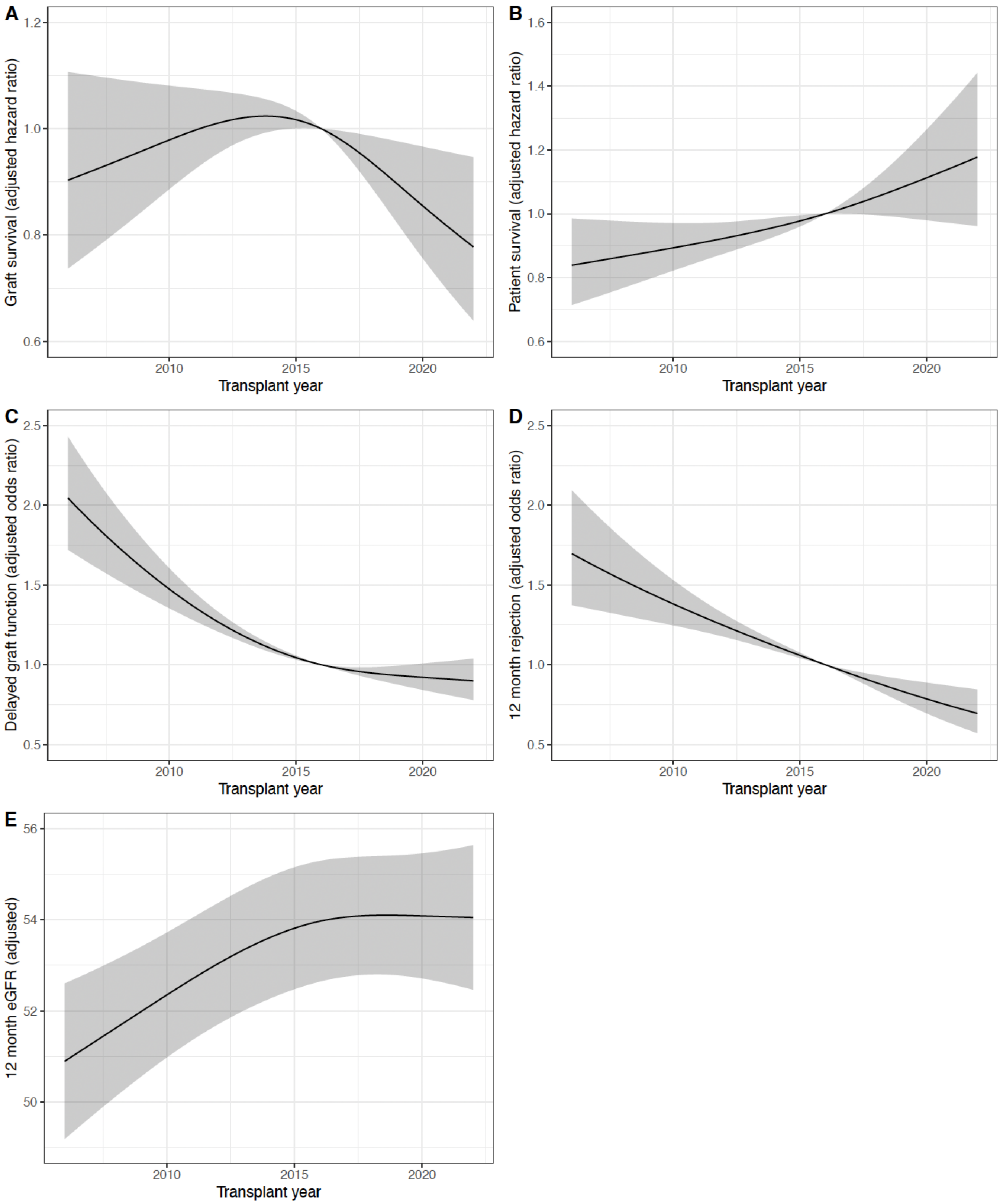

Figure S3: Association between transplant year and outcomes in deceased-donor cohort. Adjusted associations between transplant year and post-transplant outcomes, derived from multivariable models including donor, transplant and recipient factors. All models assume covariates held at their median (for continuous variables) or most common value (for categorical variables). A) Adjusted HR for graft survival (full model in Table S6). B) Adjusted hazard ratio for patient survival (full model in Table S8). C) Adjusted odds ratio for delayed graft function (full model in Table S10). D) Adjusted odds ratio for acute rejection within 12-months post-transplant (full model in Table S12). E) Predicted 12-month estimated glomerular filtration rate (full model in Table S14). Shaded areas present 95% confidence intervals.

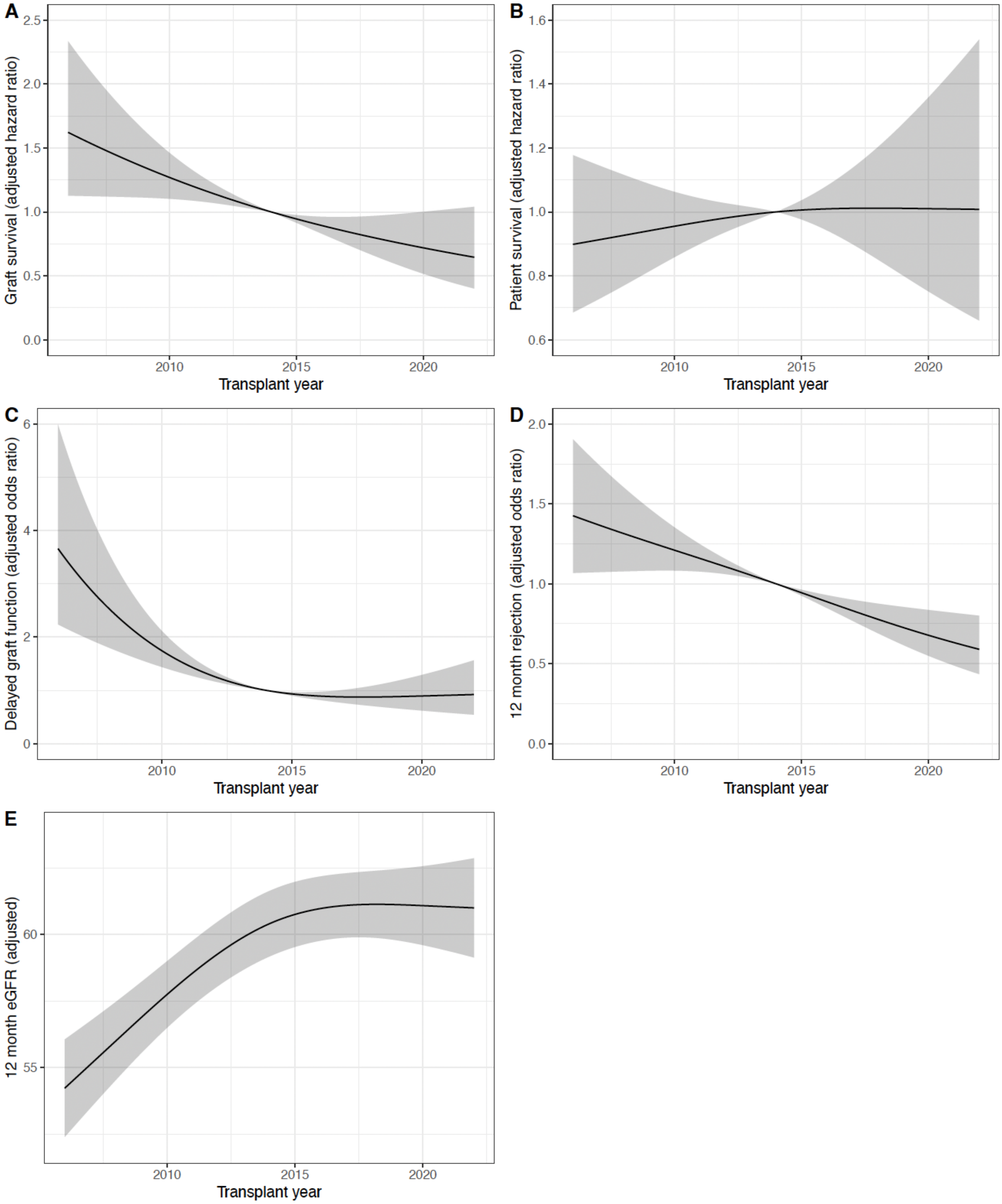

Figure S4: Association between transplant year and outcomes in live-donor cohort. Adjusted associations between transplant year and post-transplant outcomes, derived from multivariable models including donor, transplant and recipient factors. All models assume covariates held at their median (for continuous variables) or most common value (for categorical variables). A) Adjusted HR for graft survival (full model in Table S7). B) Adjusted hazard ratio for patient survival (full model in Table S9). C) Adjusted odds ratio for delayed graft function (full model in Table S11). D) Adjusted odds ratio for acute rejection within 12-months post-transplant (full model in Table S13). E) Predicted 12-month estimated glomerular filtration rate (full model in Table S15). Shaded areas present 95% confidence intervals.

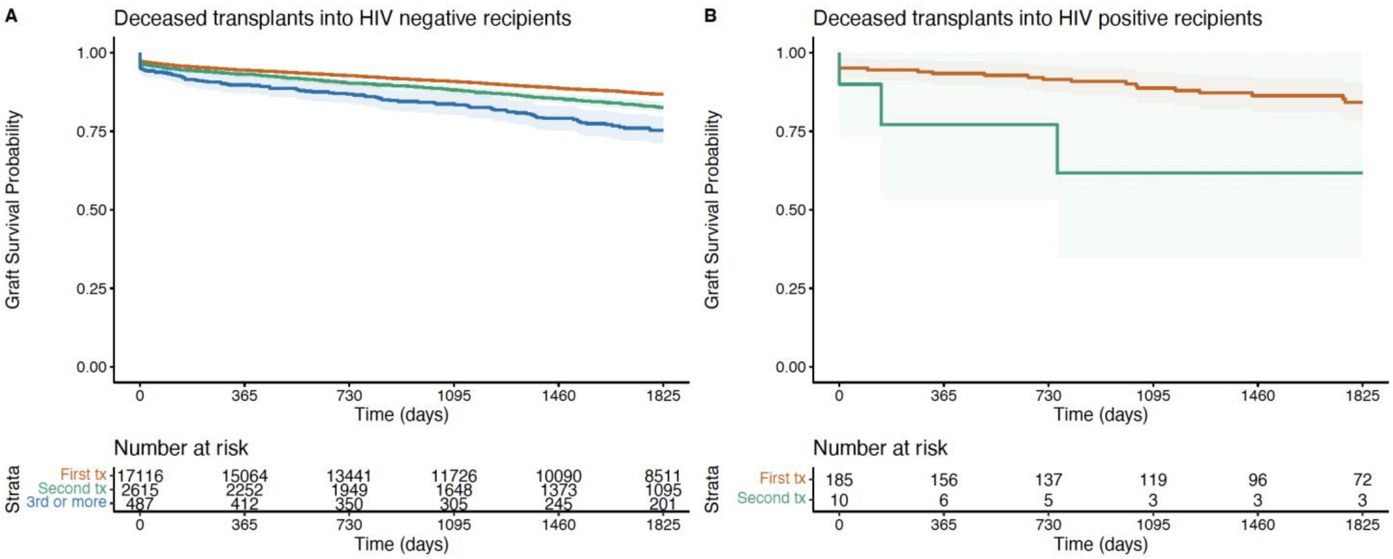

Figure S5: Kaplan-Meier plots showing 5-year graft survival in the full deceased donor cohort, stratified by kidney re-transplant status. A) HIV-negative recipients. B) HIV-positive recipients. Shaded areas indicate 95% confidence intervals. Number at risk is shown beneath each plot.
